# Genome-wide identification and expression pattern analysis of lipoxygenase gene family in banana

**DOI:** 10.1038/s41598-021-89211-6

**Published:** 2021-05-11

**Authors:** Fan Liu, Hua Li, Junwei Wu, Bin Wang, Na Tian, Jiapeng Liu, Xueli Sun, Huan Wu, Yuji Huang, Peitao Lü, Chunzhen Cheng

**Affiliations:** 1 College of Horticulture, Fujian Agriculture and Forestry University, Fuzhou, 350002 China; 2 College of Horticulture, Shanxi Agricultural University, Taigu, 030801 China; 3 College of Life Sciences, South China Agricultural University, Guangzhou, 510000 China

**Keywords:** Biotechnology, Molecular biology

## Abstract

The LOX genes have been identified and characterized in many plant species, but studies on the banana LOX genes are very limited. In this study, we respectively identified 18 MaLOX, 11 MbLOX, and 12 MiLOX genes from the *Musa acuminata*, *M. balbisiana* and *M. itinerans* genome data, investigated their gene structures and characterized the physicochemical properties of their encoded proteins. Banana *LOXs* showed a preference for using and ending with G/C and their encoded proteins can be classified into 9-LOX, Type I 13-LOX and Type II 13-LOX subfamilies. The expansion of the *MaLOXs* might result from the combined actions of genome-wide, tandem, and segmental duplications. However, tandem and segmental duplications contribute to the expansion of *MbLOXs*. Transcriptome data based gene expression analysis showed that *MaLOX1*, *4*, and *7* were highly expressed in fruit and their expression levels were significantly regulated by ethylene. And 11, 12 and 7 *MaLOXs* were found to be low temperature-, high temperature-, and *Fusarium oxysporum* f. sp. *Cubense* tropical race 4 (*Foc*TR4)-responsive, respectively. *MaLOX8*, *9* and *13* are responsive to all the three stresses, *MaLOX4* and *MaLOX12* are high temperature- and *Foc*TR4-responsive; *MaLOX6* and *MaLOX17* are significantly induced by low temperature and *Foc*TR4; and the expression of *MaLOX7* and *MaLOX16* are only affected by high temperature. Quantitative real-time PCR (qRT-PCR) analysis revealed that the expression levels of several *MaLOXs* are regulated by MeJA and *Foc*TR4, indicating that they can increase the resistance of banana by regulating the JA pathway. Additionally, the weighted gene co-expression network analysis (WGCNA) of *MaLOXs* revealed 3 models respectively for 5 (*MaLOX7*-*11*), 3 (*MaLOX6*, *13*, and *17*), and 1 (MaLOX*12*) MaLOX genes. Our findings can provide valuable information for the characterization, evolution, diversity and functionality of MaLOX, MbLOX and MiLOX genes and are helpful for understanding the roles of *LOXs* in banana growth and development and adaptations to different stresses.

## Introduction

Lipoxygenases (LOXs, EC:1.13.11.12), non-heme iron-containing oxygenases catalyzing the oxygenation of polyunsaturated fatty acids to produce fatty acid hydroperoxides, play important roles in various physiological progresses such as growth and development, signal transduction, abiotic and biotic stress responses of plants^1^. The N-terminal and C-terminal of LOX respectively contains a conserved PLAT/LH2 (polycystin-1, lipoxygenase, alpha-toxin/lipoxygenase homology) domain and a typical LOX domain^2^. The PLAT/LH2 domain functions in mediating the interaction between enzyme and biological membranes^3^. While the LOX domain, existing a histidine (His)-rich region consisted of [His-(X)4-His-(X)4-His-(X)17-His-(X)8-His], is critical for the iron coordination, substrate binding and enzyme activity^4^. According to their oxygenation sites on the fatty acid carbon chain, LOXs can be further divided into 9-LOX and 13-LOX^5^. Moreover, 13-LOXs can be further classified into type I 13-LOX and type II 13-LOX subgroups according to the absence (Type I) or presence (Type II) of chloroplast transit peptides in their N-terminals^6^.

*LOXs* are ubiquitously distributed in plants and have been isolated from a variety of plant species, such as *Arabidopsis*^7^, rice^7^, tomato^8^, poplar^9^, tea^10^, cotton^11^, peach^12^, and radish^13^. The expression of plant *LOXs* have been proved to be regulated by some phytohormones and pathogens. For instance, the expression of Arabidopsis *AtLOX1* was abscisic acid and JA inducible^14^, and the rice *OsLOX3* was MeJA and *Magnaporthe Grisea* inducible^15^. Their diverse functions during plant growth and developmental and stress response processes have also been experimentally confirmed in various plant species. Arabidopsis *AtLOX3* and *AtLOX4* double mutant plants showed developmental dysfunctions of higher plant height and increased inflorescence shoots and flowers^16^. *AtLOX2* and *AtLOX6* are found to be involved in wound induced JA synthesis in leaves^17,18^. Transgenic plants overexpressing rice *OsLOX2* showed shortened seed germination time^19^. Kiwifruit *AdLOXs* were involved in the formation of fruit aroma^20^. Silencing of *CaLOX2* in pepper plants resulted in decreased JA accumulation and reduced thrips resistance^21^. Transgenic tomato plants overexpressing the tomato lipoxygenase D (TomLoxD) gene resulted in enhanced wound-induced JA biosynthesis and increased *Helicoverpa armigera* and *Botrytis cinerea* resistance^22^. Transgenic *Arabidopsis* plant overexpressing persimmon *DkLOX3* showed increased salt tolerance and disease resistance^23^*.*

Banana, as one of the most important and popular fruit, is an herbaceous perennial plant belonging to Musa family. Cultivated banana is generally low in stress resistance and is susceptible to external environmental stresses such as low temperature and *Fusarium* wilt^24^. There are also several reports on the expression patterns of some banana *LOXs* using omic techniques, and their roles in banana responses to high temperature, low temperature and *Fusarium* wilt have been described^25–28^. Given that *LOXs* are vital for plant growth and stress resistance and different *LOX* members’ functions varied, it is of great importance to analyze the LOX gene family from whole genome level for the clarification of their diverse potentials in banana. In the present study, whole genome wide LOX gene family identification was performed based on the *M. acuminata*, *M. balbisiana* and *M. itinerans* genome data. Totally, we identified 18 *MaLOX*, 11 *MbLOX*, and 12 *MiLOX* family members, which were then subjected to series of bioinformatics analysis to show the chromosome location, gene structure and gene duplication events of LOX genes and to reveal the physiological and biochemical characteristics, subcellular localization, and phylogenetic relationship of their encoded proteins. Moreover, the expression patterns of *MaLOXs* were investigated using quantitative real time PCR (qRT-PCR) and transcriptome data. Our preliminary results can extend the knowledge of banana LOX gene family and can provide insights into their roles in banana growth and development and stress responses.

## Materials and methods

### Plant materials

In our previous study, ‘Tianbaojiao’ banana (*Musa* spp., Cavendish, AAA group) plantlets were used for transcriptome profiling to show the transcriptome changes caused by 4 ℃ low temperature in leaves of four-leaf stage plantlets, by 45 ℃ high temperature in leaves of five-stage plantlets, and by *Foc*TR4 inoculation in banana roots. Moreover, transcriptome changes of natural ripening and ethylene treated ‘Tianbaojiao’ banana fruits at 0, 1, 3, and 5 days were also compared. Moreover, to show the influence of MeJA treatment on the expression of banana LOXs, ‘Brizil’ banana (*Musa acuminata* cv. Brazil) plantlets at six-leaf stage were exposed to 100 mM MeJA solution (containing 0.02% (v/v) Tween 20) treatment^9^, treated leaves were sampled at 0, 6, 12, 24 h after MeJA treatment. In addition, in order to further explore the expression of *MaLOXs* in response to *Foc*TR4 treatment, ‘Zhongjiao No.3’ banana (*Musa acuminata* cv. Brazil) plantlets at six-leaf stage were inoculated with 1 × 10^7^/mL *Foc*TR4 spore suspension according to the inoculation method described by Wang et al.^29^. Roots were collected 0 day, 4 days, 2 weeks, and 4 weeks after treatment. Banana plantlets showed no visible symptom in corm until 4 weeks after *Foc*TR4 inoculation. All samples were immediately frozen in liquid nitrogen and stored at − 80 °C for further use. For qRT-PCR analysis, three independent replicates were used for each time point of MeJA and *Foc*TR4 treatments. All the banana materials used in this research were harvested from cultivated varieties (‘Tianbaojiao’ banana is a famous traditional cultivar in Tianbao county, Fujian province, China. ‘Brazil’ is one of the most popular banana variety in the world and ‘Zhongjiao No.3’ is a new banana variety selected from ‘Brazil’ by Institute of fruit science, Guangdong Agricultural Academy), and their collections complied with relevant institutional, national, and international guidelines and legislation.

### Identification of banana LOX genes

The genomic DNA, CDS, and protein sequence files of *M. acuminata* var. DH-Pahang, *M. balbisiana* var. DH PKW and *M. itinerans* var. Yunnan were downloaded from the banana genome databases (https://banana-genome-hub.southgreen.fr/). HMMER3.0 software was used to search against the banana protein sequences using The Hidden Markov Model file of Lipoxygenase (PF00305) downloaded from the Pfam database (http://pfam.xfam.org/) with E-value ≤ 1 × 10^–5^ to obtain candidate LOX proteins, which were further submitted to conserved domain database (CDD, https://www.ncbi.nlm.nih.gov/cdd) for the confirmation of the existence of the lipoxygenase and PLAT/LH2 domains^10^. Sequences without Lipoxygenase domain and/or PLAT/LH2 domain were removed. The remaining banana LOXs are named sequentially according to the chromosomal location of their corresponding genes. ExPASy (https://web.expasy.org/protparam/) was used to analyze the basic physicochemical properties of LOX proteins. Chloroplast transit peptide and subcellular localization were predicted by ChloroP 1.1 Server (http://www.cbs.dtu.dk/services/ChloroP/) and WoLF PSORT (https://wolfpsort.hgc.jp/). The global sequence alignment program Needle (https://www.ebi.ac.uk/Tools/psa/emboss_needle/) in the EMBOSS tool was used to perform pairwise alignment of protein sequences to determine the similarity and identity between LOX members. Gene structure of banana *LOXs* was drawn by GSDS (http://gsds.cbi.pku.edu.cn/). The conserved motifs of LOXs (20 maximum number of motifs) were analyzed using MEME suite (http://meme-suite.org/tools/meme) and visualized using TBtools software^30^. The CodonW software (version 1.4.2, http://codonw.sourceforge.net/) was used to calculate the effective number of codons (ENC), codon adaptation index (CAI), relative synonymous codon usage (RSCU), and other codon preference parameters^6^.

### Phylogenetic analysis

The LOX protein sequences of *Arabidopsis thaliana*, rice, tomato, poplar and some other plants were downloaded from TAIR (https://www.arabidopsis.org/)^7^, RGAP (http://rice.plantbiology.msu.edu/)^7^, SGN tomato (https://solgenomics.net/)^8^, Phytozome (http://www.phytozome.net/), and NCBI (http://www.ncbi.nlm.nih.gov/)^9^, respectively. After domain verification using CDD, OsLOX2, OsLOX9, OsLOX14, GmLOX2, and PvLOX2c without incomplete Lipoxygenase and PLAT/LH2 domains were removed. Multiple sequence alignment was performed using Muscle software, and phylogenetic tree was constructed by Neighbor-joining method using MEGA 6.06 (Possion mode, complete deletion, and 1000 bootstrap values) and was visualized using EvolView (https://www.evolgenius.info/evolview/).

### Chromosome location and gene duplication analysis

Blast software (version 2.10.0, https://blast.ncbi.nlm.nih.gov/Blast.cgi) was used to perform self-alignment and pairwise alignment analysis of LOX proteins (E-value ≤ 1 × 10^–10^). The intra/inter-species gene collinear relationship of the LOX family was analyzed by using MCScanX (version 0.8, http://chibba.pgml.uga.edu/mcscan2/)^31^. According to the chromosomal location information, the Circos software (version 0.69-9, http://circos.ca/) was used to visualize the syntenic relationships between banana LOXs and LOXs from other plant species^32^. KaKs_Calclator 2.0 software (https://sourceforge.net/) was used to estimate synonymous (Ks) and nonsynonymous (Ka) substitution rates^33^. For the timing of duplication events, the formula: T = Ks/2λ × 10^–6^ Mya was used to calculate divergence time (T) in millions of years (Mya), where λ = 4.5 × 10^− 9^ represented the evolution rate of *Musa*^34^.

### Analysis of *cis*-acting elements and transcription factor binding sites in the promoters of banana LOX genes

The 1500 bp upstream of the start codon of each banana *LOX* gene was extracted from the banana genome database. Due to the presence of large numbers of CTT repeat sequences on *MaLOX5* promoter region from the genome data, PCR was used to verify its true sequence. It was found that CTT repeat sequences were absent, thus the corrected sequence was used for subsequent analysis. The *cis*-acting elements of the promoter were predicted using the PlantCARE (http://bioinformatics.psb.ugent.be/webtools/plantcare/html/). PlantTFDB (http:// planttfdb.cbi.pku.edu.cn/) was used to predict the transcription factor binding sites (TFBSs) on promoters with the parameter set of p-value ≤ 1e^−6^. The promoter regions were partitioned to proximal promoter region (500 bp upstream), median promoter region (501–1000 bp upstream) and distal promoter region (1001–1500 bp upstream).

### Gene expression analysis using transcriptome data and qRT-PCR

The expression patterns of banana LOX genes under low temperature, high temperature and *Foc*TR4 treatments were analyzed using our previous transcriptome data. The expression values of banana LOX family genes were extracted from the transcriptome data, and heatmap was drawn using HemI1.0 software (http://hemi.biocuckoo.org/). qRT-PCR was used to show the expression patterns of all the banana LOX genes under JA treatment. Total RNA was extracted using RNAprep Pure Plant Kit (TIANGEN, China) according to the manufacturer’s instructions. A total of 1 μg RNA was used for cDNA synthesis using PrimeScript™ RT reagent Kit with gDNA Eraser (Perfect Real Time) (Takara, China). CDNA was diluted tenfold for subsequent experiments. The PCR reaction conditions used were 95 °C for 30 s, 95 °C for 5 s, and 60 °C for 34 s (40 cycles). Relative gene expression levels were determined using the 2^-∆∆Ct^ method by using *MaCAC* as an internal reference^35^. Primers were designed using Oligo 7.0, and their specificity was checked using information obtained from the NCBI website. All primers used in this study are listed in Supplemen Table S1. Statistical analysis and figure drawing were conducted using SPSS 25.0 and GraphPad Prism 6.0 software, respectively.

### Weighted gene co-expression network analysis (WGCNA)

Genes with FPKM value greater than 10 in at least one RNA-Seq sample were subjected to WGCNA (version 1.68) analysis to construct and identify co-expressed gene clusters with *MaLOXs*^36^. The parameters were set as follows: The optimal β (soft thresholding power) value was 12; the minModuleSize was 30 and the mergeCutHeight was 0.25. Finally, we extracted the co-expression network of all *MaLOXs* and filtered out the edges with weights below 0.4. We visualized the network connections using the Cytoscape (version 3.8.0, https://cytoscape.org/) program^37^. The functional enrichment analysis of *MaLOXs* and co-expressed genes was performed using Gene Ontology (GO), and Kyoto Encyclopedia of Genes and Genomes (KEGG) databases.

## Results

### Identification and characterization of banana LOX gene family members

Totally, 18, 11, and 12 LOX genes were identified from *M. acuminata*, *M. balbisiana,* and *M. itinerans* genome, respectively (Table 1, Supplementary Table S2). According to their chromosomal location information, the 18 *MaLOXs* were defined as *MaLOX1-MaLOX18*, respectively. Among these *MaLOXs*, *MaLOX5* had two transcripts, which was named as *MaLOX5a* and *MaLOX5b*, respectively. *MbLOXs* and *MiLOXs* were named in concordance with their *MaLOXs* homologous (Supplementary Figure S1).

**Table 1:** The information of LOX gene family in banana.

Species	Gene ID	Transcript ID	Gene name	Chromosome location	CDS/bp	Size/aa	Molecular weight/kD	PI	Chloroplast transit peptides	Subcellular localization
*Musa acuminata*	Ma01_g16400	Ma01_t16400.1	*MaLOX1*	chr01:11878658..11882747 (+)	2619	872	98.62	6.03	−	CP
	Ma01_g18020	Ma01_t18020.1	*MaLOX2*	chr01:13322958..13326238 (−)	2544	847	95.49	6.33	−	CP
	Ma01_g18040	Ma01_t18040.1	*MaLOX3*	chr01:13344016..13347394 (−)	2544	847	95.19	6.31	−	CP
	Ma01_g18060	Ma01_t18060.1	*MaLOX4*	chr01:13358464..13361737 (−)	2586	861	96.38	6.05	−	CP
	Ma02_g07800	Ma02_t07800.1	*MaLOX5a*	chr02:18320819..18324221 (−)	2082	693	77.57	9.53	−	CP
		Ma02_t07800.2	*MaLOX5b*		2562	853	97.04	7.00	−	CP
	Ma03_g07770	Ma03_t07770.1	*MaLOX6*	chr03:5495340..5499190 (−)	2745	914	10.30	7.71	Y	Chl
	Ma03_g11520	Ma03_t11520.1	*MaLOX7*	chr03:8935641..8940695 (−)	2724	907	10.20	6.37	Y	Chl
	Ma06_g26840	Ma06_t26840.1	*MaLOX8*	chr06:28772817..28775490 (+)	2061	686	76.33	5.90	−	ER
	Ma06_g26850	Ma06_t26850.1	*MaLOX9*	chr06:28828834..28832329 (+)	2622	873	97.33	5.78	−	CP
	Ma06_g26870	Ma06_t26870.1	*MaLOX10*	chr06:28849493..28860078 (+)	3396	1131	126.29	6.26	−	CP
	Ma06_g26890	Ma06_t26890.1	*MaLOX11*	chr06:28883457..28886953 (+)	2622	873	97.34	6.00	−	ER
	Ma06_g30170	Ma06_t30170.1	*MaLOX12*	chr06:31521537..31528304 (−)	2739	912	102.66	7.37	Y	Chl
	Ma08_g23400	Ma08_t23400.1	*MaLOX13*	chr08:36811891..36815961 (+)	2736	911	102.55	6.41	Y	Chl
	Ma09_g12090	Ma09_t12090.1	*MaLOX14*	chr09:8177556..8181866 (−)	2712	903	101.93	6.31	Y	Chl
	Ma09_g15420	Ma09_t15420.1	*MaLOX15*	chr09:10750253..10754168 (−)	2733	910	102.29	6.61	Y	Chl
	Ma09_g19130	Ma09_t19130.1	*MaLOX16*	chr09:19917117..19920868 (−)	2856	951	107.51	6.31	−	CP
	Ma09_g19140	Ma09_t19140.1	*MaLOX17*	chr09:19917453..19921016 (−)	2568	855	96.01	5.72	−	CP
	Ma10_g17560	Ma10_t17560.1	*MaLOX18*	chr10:28903758..28908078 (−)	2760	919	102.84	8.15	Y	CP
*Musa balbisiana*	Mba01_g25910	Mba01_g25910.1	*MbLOX1*	Bchr01:20001697…20005726 (+)	2610	869	98.35	6.05	−	CP
	Mba03_g07670	Mba03_g07670.1	*MbLOX6*	Bchr03:5604842…5608862 (−)	1998	665	75.15	9.40	Y	Chl
	Mba06_g26190	Mba06_g26190.1	*MbLOX10*	Bchr06:31900379…31903722 (+)	2580	859	96.02	5.68	−	CP
	Mba06_g26200	Mba06_g26200.1	*MbLOX9*	Bchr06:31926218…31929597 (+)	2727	908	101.59	6.20	−	CP
	Mba06_g26210	Mba06_g26210.1	*MbLOX8*	Bchr06:31964229…31967505 (+)	2670	889	99.00	5.97	−	Chl
	Mba06_g26220	Mba06_g26220.1	*MbLOX11*	Bchr06:31978370…31981642 (+)	2682	893	99.41	6.41	−	Chl
	Mba08_g23020	Mba08_g23020.1	*MbLOX13*	Bchr08:36835632…36839686 (+)	2742	913	102.96	6.56	Y	Chl
	Mba09_g11450	Mba09_g11450.1	*MbLOX14*	Bchr09:8300639…8304936 (−)	2172	723	82.14	6.28	Y	Chl
	Mba09_g14640	Mba09_g14640.1	*MbLOX15*	Bchr09:10993723…10997549 (−)	2286	761	85.39	7.64	Y	Chl
	Mba09_g18010	Mba09_g18010.1	*MbLOX16*	Bchr09:16550622…16556265 (+)	2568	855	95.98	5.68	−	CP
	Mba10_g15430	Mba10_g15430.1	*MbLOX18*	Bchr10:32779595…32784030 (−)	2658	885	98.85	8.38	Y	CP
*Musa itinerans*	Mi_g004153	Mi_g004153	*MiLOX1*	scaffold1338:259261…263079 (+)	2532	843	94.99	6.12	−	CP
	Mi_g014015	Mi_g014015	*MiLOX5*	scaffold2542:171670…174847 (+)	2472	823	93.78	6.62	−	CP
	Mi_g017218	Mi_g017218	*MiLOX18*	scaffold3004:214416…218434 (−)	2712	903	101.07	8.45	−	CP
	Mi_g017373	Mi_g017373	*MiLOX13*	scaffold3031:97765…101524 (−)	2631	877	98.92	6.35	Y	Chl
	Mi_g018964	Mi_g018964	*MiLOX10*	scaffold34:234414…237513 (−)	2535	845	94.59	5.89	−	CP
	Mi_g021392	Mi_g021392	*MiLOX15*	scaffold406:321838…325445 (+)	2619	873	98.22	6.79	Y	CP
	Mi_g027370	Mi_g027370	*MiLOX16*	scaffold647:55264…58996 (−)	2847	949	107.52	6.57	−	CP
	Mi_g027442	Mi_g027442	*MiLOX7*	scaffold649:208052…213703 (−)	2541	847	95.23	6.92	Y	Chl
	Mi_g027551	Mi_g027551	*MiLOX14*	scaffold655:11648…15808 (+)	2532	843	95.23	6.53	Y	Chl
	Mi_g028690	Mi_g028690	*MiLOX12*	scaffold7089:49982…56244 (−)	2613	870	97.84	7.36	Y	Chl
	Mi_g029482	Mi_g029482	*MiLOX6*	scaffold768:345315…348813 (−)	2487	829	93.83	6.66	−	Chl
	Mi_g030630	Mi_g030630	*MiLOX4*	scaffold829:57403…60534 (−)	2568	855	96.06	5.95	−	CP

Chloroplast transit peptides: Y: yes. Subcellular localization: CP: Cytoplasm; Chl: Chloroplast; ER: Endoplasmic reticulum.

The CDS length of *MaLOXs* ranged from 2061 to 3396 bp. Their deduced proteins contained 686–1131 amino acids (aa) with theoretical isoelectric points ranged from 5.72 to 9.53. The molecular weight of MaLOXs ranged from 76.33 to 126.29 kD. MbLOX proteins contained 665 to 913 aa with molecular weight ranged from 75.15 to 102.96 kD. MiLOX proteins contained 823 to 949 aa, with molecular weight ranged from 93.78 to 107.52 kD. Their theoretical isoelectric point ranged from 5.68 to 9.40 and from 5.89 to 8.45 for MbLOXs and MiLOXs, respectively. Chloroplast transit peptides were identified in 7 MaLOX (MaLOX6-7, 12–15, and 18), 5 MbLOX (MbLOX6, 13, 14, 15, and 18), and 5 MiLOX (MiLOX7, 12, 13, 14, and 15) members, respectively. The MaLOXs were predicted to located in different cell parts, most of which were cytoplasm located, while MaLOX6-7 and 12–15 were chloroplast located. Six of 11 MbLOX proteins were located in the cytoplasm, and 5 proteins were located in the chloroplast. In addition, 7 MiLOXs were located in the cytoplasm and 5 in the chloroplast.

**Figure 1:**
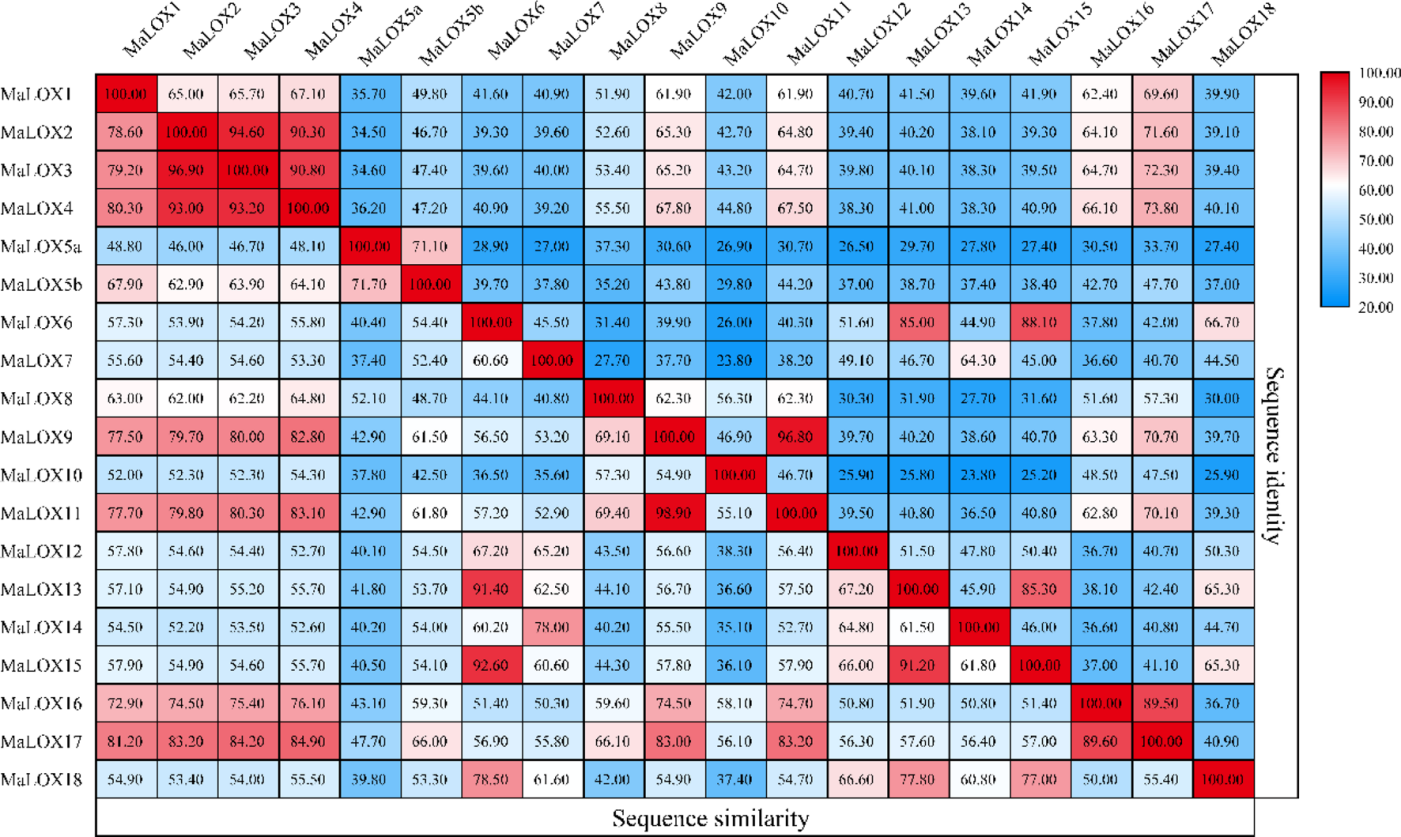
Sequence identities and similarities (%) among the *MaLOXs*.

Protein sequence alignment result revealed that the sequence similarity among MaLOXs ranged from 35.10 to 98.90%, and the sequence identity ranged from 23.80 to 96.80% (Fig. 1). The similarity and identity between MaLOX5a and other members are relatively low, and MaLOX9 and MaLOX11 showed the highest similarity and identity, while the similarity and identity between MaLOX10 and MaLOX14 was the lowest (Fig. 1). Besides, the sequence similarity among MbLOXs and MiLOXs was 37.60–95.2% and 47.30–91.00%, and sequence identity was 27.60–93.70% and 33.90–84.70%, respectively (Supplementary Figures S2, S3).

### Phylogenetic relationship of banana LOX protein family

**Figure 2:**
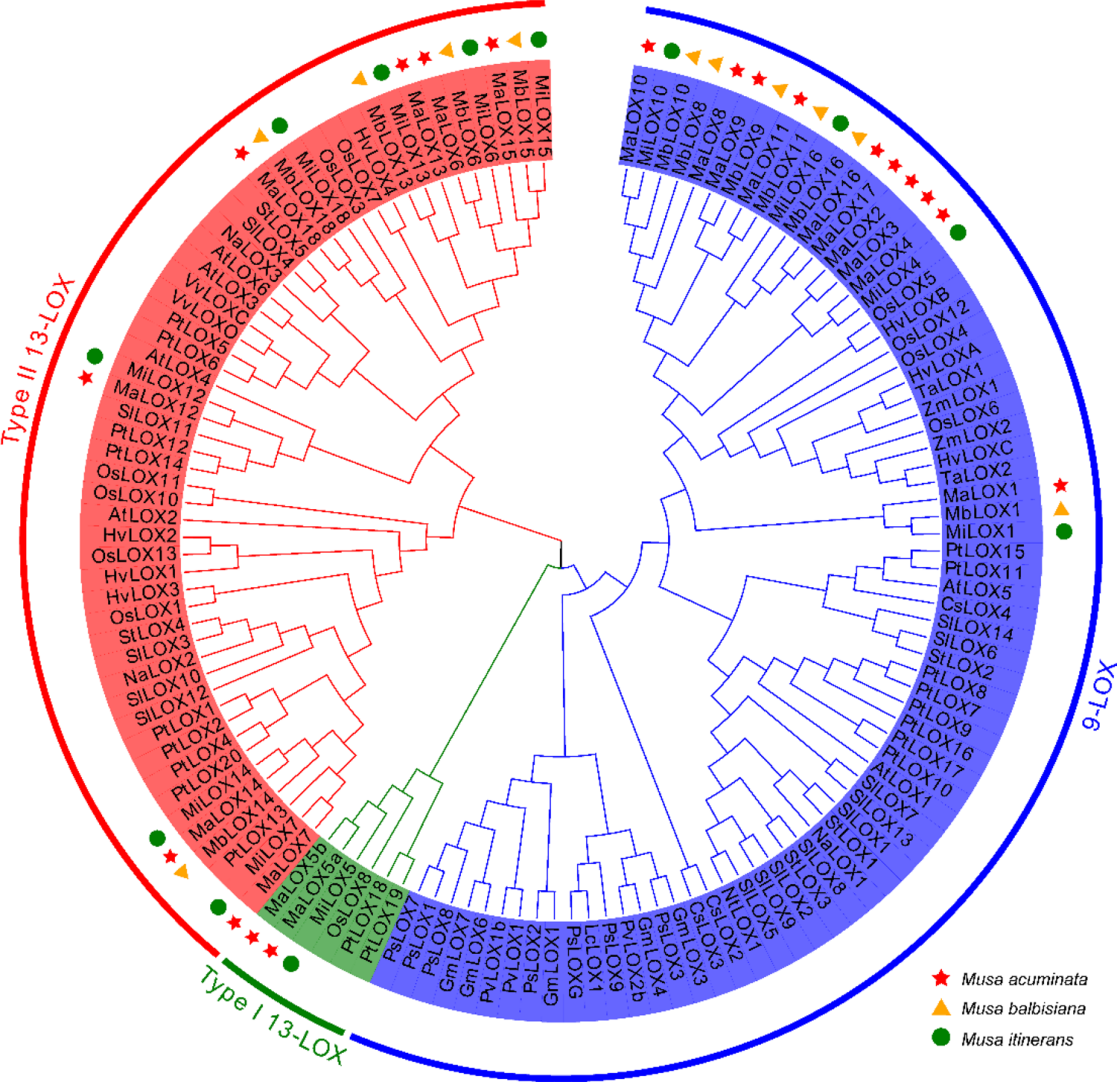
Phylogenetic tree of LOX proteins from *M. acuminata*, *M. balbisiana*, *M. itinerans* and some other plant species.

To determine the phylogenetic relationship among MaLOXs, MbLOXs, and MiLOXs, the LOX protein sequences from *Arabidopsis* (6), rice (11), tomato (14), poplar (19), banana (42) and other plants (41) were used for phylogenetic analysis. All LOX proteins could be classified into two subfamilies, 9-LOX and 13-LOX. And 13-LOX can be further divided into Type I and Type II (Fig. 2). The 9-LOX subfamily includes 10 MaLOXs (MaLOX1-4, 8–11, 16, and 17), 6 MbLOXs (MbLOX1, 8, 9, 11, and 16), and 4 MiLOXs (MiLOX1, 4, 10, and 16), respectively. Seven MaLOXs, 5 MbLOXs and 7 MiLOXs belong to Type II 13-LOX subfamily. MaLOX5a, MaLOX5b, and MiLOX5 belong to the Type I 13-LOX subfamily, and this subfamily only contains banana, rice and poplar LOXs.

### Analysis of gene structure and conserved domain

**Figure 3:**
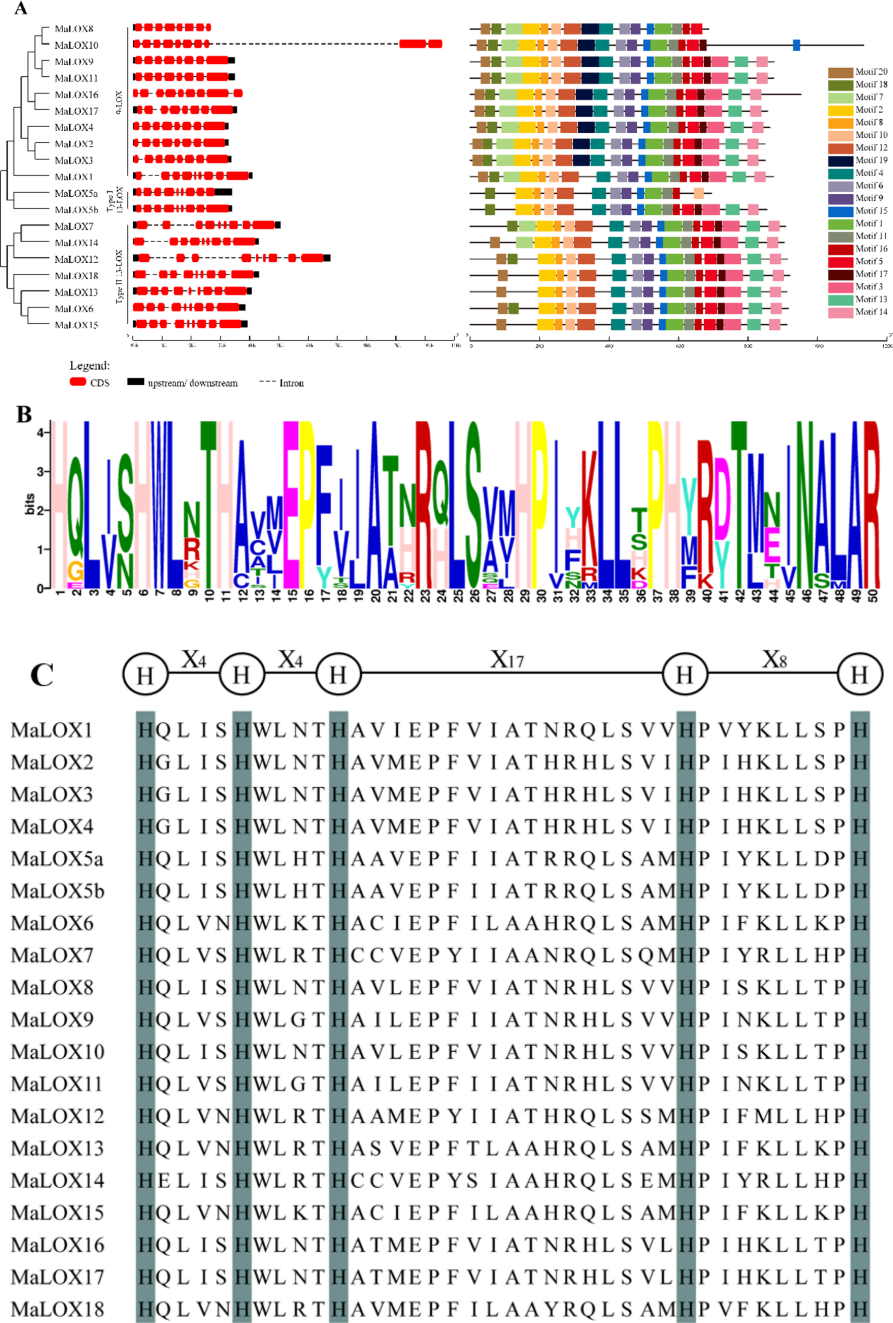
Gene structures and conserved motifs (**A**), Motif 1 sequence (**B**) and the 38 conserved residues of MaLOX proteins of *MaLOXs* or their encoded proteins. The dark color in C shows highly conserved histidine (His).

GSDS was used to show the gene structure diagram of *MaLOXs*, *MbLOXs*, and *MiLOXs*. As shown in Fig. 3A, *MaLOX*s have 8–10 exons, of which *MaLOX10* has the largest numbers of exons. The exons of 9-LOX subfamily genes are very similar in length and distribution, suggesting that this subfamily may originate from the same ancestor gene. Most *MaLOX* members have gDNA lengths between 3 and 5 kb, except *MaLOX10* and *MaLOX12*, whose gDNA length is about 11 kb and 7 kb, respectively. *MbLOXs* and *MiLOXs* have similar gene structures with *MaLOXs. MbLOXs* contain 6–10 exons, and *MiLOXs* have 8–10 exons (Supplementary Figures S4A, S5A). In addition, most banana *LOX* genes within the same subfamily presented similar exon–intron distribution patterns.

Conserved motif analysis showed that most banana LOXs contained similar types and arrangements of conserved motifs (Fig. 3A, Supplementary Figures S4A, S5A). Eleven conserved motifs (motif 1, 2, 4, 6, 8–12, 15 and 16) were found in all MaLOXs. In addition to the common motifs, the 9-LOX subfamily proteins also contain motifs 5, 7, 18, and 20, and the Type II 13-LOX subfamily also contains motifs 3, 5, 13–14, and 17. MbLOXs and MiLOXs have similar conserved motifs with MaLOXs. The histidine (His)-rich Motif 1, plays an important role in the biological activity of lipoxygenase, is highly conserved among banana LOX family members (Fig. 3B, Supplementary Figures S4B, S5B). A typical domain of the banana LOXs is consisted of 38 amino acids of [His-(X)4-His-(X)4-His-(X)17-His-(X)8-His] (Fig. 3C, Supplementary Figures S4C, S5C).

### Chromosome location and gene duplication

**Figure 4:**
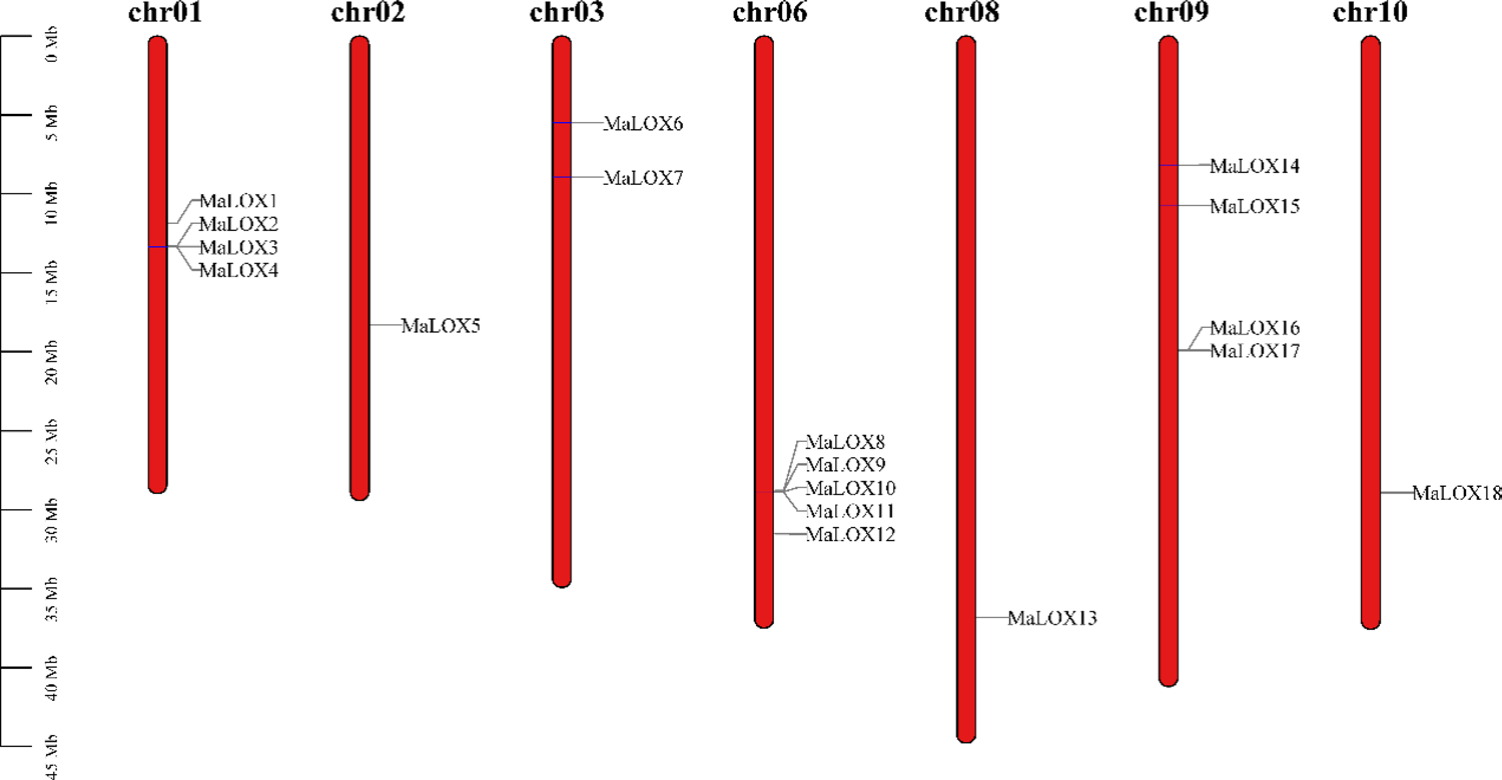
Chromosome localization of MaLOX genes.

As shown in Fig. 4, MaLOX genes are randomly and unevenly distributed on 7 chromosomes (chr). The highest number of *MaLOXs* was observed in chr06, with 5 members, follow by chr01, 03 and 09 with 4, 2, and 4 members, respectively. Eleven MbLOX genes were located on 6 of the 11 chromosomes (Bchr) and exhibited uneven distributions (Supplementary Figure S6). Bchr06 contained the highest number of MbLOX genes (4, 36.36%), followed by Bchr09 (3, 27.27%), while minimum genes were distributed on Bchr01, 03, 08, and 10 (1, 9.09%). *M. itinerans* genome was only assembled to the scaffold level (S). The 12 MiLOX genes are identified from 12 different scaffolds (S1338, S2542, S3004, S3031, S34, S406, S647, S649, S655, S7089, S768, S829) (Supplementary Figure S7).

**Figure 5:**
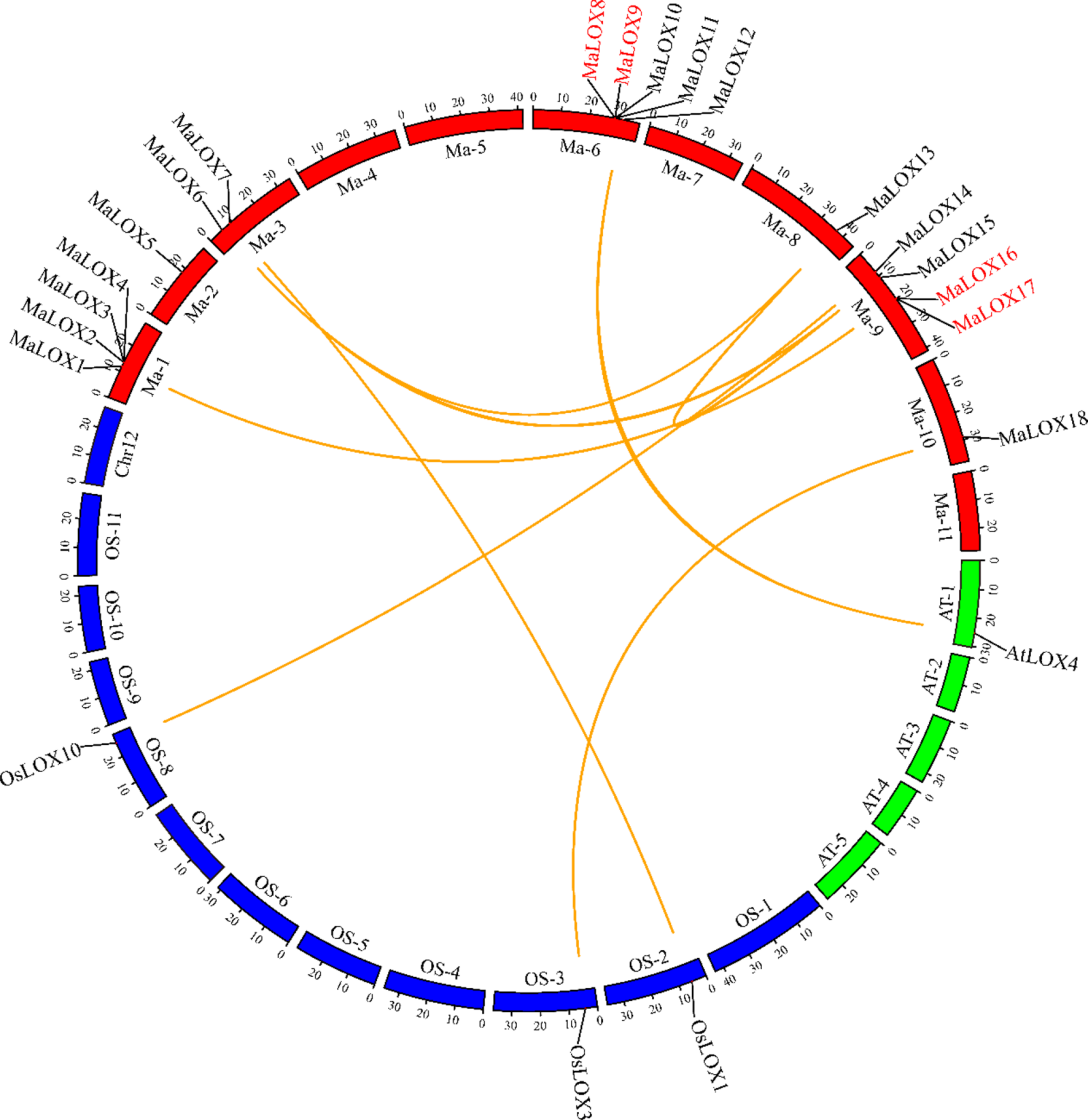
Collinear distribution of MaLOX genes. The orange line indicates the collinearity between the *MaLOXs*, and the gene names in red are tandem replication genes.

In order to explore the gene duplication events of the *LOX* family, we investigated the collinearity relationships between banana *LOXs* as well as pairwise relationships analysis of *LOXs* from *M. acuminata*, *M. balbisiana*, *M. itinerans*, *Arabidopsis*, and rice (Fig. 5; Supplementary Figures S8, S9, S10; Table 2; Supplementary Tables S3, S4). There are 2 tandem duplicate pairs (*MaLOX8/MaLOX9* and *MaLOX16/MaLOX17*) and 4 segmental duplicate pairs (*MaLOX1/MaLOX16*, *MaLOX6/MaLOX13*, *MaLOX6/MaLOX15*, and *MaLOX13/MaLOX15*) in the MaLOX gene family. MbLOX gene family has 3 tandem duplicate pairs (*MbLOX10/MbLOX9*, *MbLOX9/MbLOX8*, and *MbLOX8/MbLOX11*) and 3 segmental duplicate pairs (*MbLOX6/MbLOX13*, *MbLOX6/MbLOX15*, and *MbLOX13/MbLOX15*) (Supplementary Figure S8; Supplementary Table S3). However, MiLOX gene family does not contain any duplicated pairs (Supplementary Figure S9). In addition, three OsLOX genes had a syntenic relationship with three *MaLOXs* (*OsLOX1/MaLOX7*, *OsLOX3/MaLOX18*, and *OsLOX10/MaLOX14*) and 1 collinear pair (*AtLOX4/MaLOX12*) was identified between *M. acuminata* and *Arabidopsis*.

**Table 2:** LOX gene family intraspecific and interspecific gene replication events.

Gene name	Gene ID	Gene name	Gene ID	Ka	Ks	Ka/Ks	Duplication date /Mya	Duplication type
*MaLOX1*	Ma01_g16400	*MaLOX16*	Ma09_g19130	0.2021	0.9918	0.2038	110.20	Segmental duplication
*MaLOX6*	Ma03_g07770	*MaLOX13*	Ma08_g23400	0.0760	0.4850	0.1566	53.89	Segmental duplication
*MaLOX6*	Ma03_g07770	*MaLOX15*	Ma09_g15420	0.0596	0.3439	0.1733	38.21	Segmental duplication
*MaLOX13*	Ma08_g23400	*MaLOX15*	Ma09_g15420	0.0751	0.5039	0.1490	55.99	Segmental duplication
*MaLOX8*	Ma06_g26840	*MaLOX9*	Ma06_g26850	0.1298	0.5639	0.2302	62.65	Tandem duplication
*MaLOX16*	Ma09_g19130	*MaLOX17*	Ma09_g19140	0.0028	0.0008	3.5461	0.09	Tandem duplication
*AtLOX4*	AT1G67560	*MaLOX12*	Ma06_g30170	0.3223	2.9750	0.1083		Segmental duplication
*OsLOX1*	LOC_Os02g10120	*MaLOX7*	Ma03_g11520	0.3320	0.9242	0.3593		Segmental duplication
*OsLOX3*	LOC_Os03g08220	*MaLOX18*	Ma10_g17560	0.1624	1.0741	0.1512		Segmental duplication
*OsLOX10*	LOC_Os08g39840	*MaLOX14*	Ma09_g12090	0.3114	1.4462	0.2153		Segmental duplication

The collinearity relationships between three banana species are shown in Supplementary Figure S10. Fourteen orthologous gene pairs between *M. acuminata* and *M. balbisiana* were identified, 12 orthologous gene pairs were found between *M. acuminata* and *M. itinerans*, and 9 orthologous gene pairs existed between *M. balbisiana* and *M. itinerans*. Moreover, some LOX genes are relatively conserved between banana species. For example, *MaLOX1*, *6*, *9, 13–15*, and *18* have collinearity with their orthologs in *M. balbisiana* and *M. itinerans*.

To further understand whether the genes of the *LOX* family have been subjected to natural selection pressures during the evolution process and to trace the duplication time of banana *LOXs*, we calculated the ratios of nonsynonymous (Ka) versus synonymous (Ks) mutation of orthologous gene pairs. As shown in Table 2, Supplementary Table S3, and Supplementary Table S4, the Ka/Ks ratios of *MaLOX16/MaLOX17* is more than 1, which may have experienced strong positive selection. In addition, the Ka/Ks ratios of other duplicate pairs less than 1, suggesting that these pairs have undergone purifying selection pressure during evolution.

Based on the divergence rate of 4.5 × 10^–9^ synonymous mutations per synonymous site year proposed for banana, we estimated the time of occurrence of duplicating events of the paralogous *LOX* gene pairs. The results showed that *MaLOX1/MaLOX16* and *MaLOX16/MaLOX17* occurred at about 110.20 and 0.09 million years ago (Mya), while other *MaLOX* gene pairs occurred between 38.21 to 62.65 Mya (Table 2). The estimated divergence time of the duplicated gene pairs of *MbLOX* family varies from 47.54 to 74.29 Mya (Supplementary Table S3). Furthermore, the replication times for syntenic genes between *MaLOX* and *MbLOX*, between *MaLOX* and *MiLOX*, and between *MbLOX* and *MiLOX* was 3.10–107.17 Mya, 2.38–70.73 Mya, and 3.39–50.98 Mya, respectively (Supplementary Table S4).

### *Cis*-acting elements prediction results of banana LOX gene promoters

**Figure 6:**
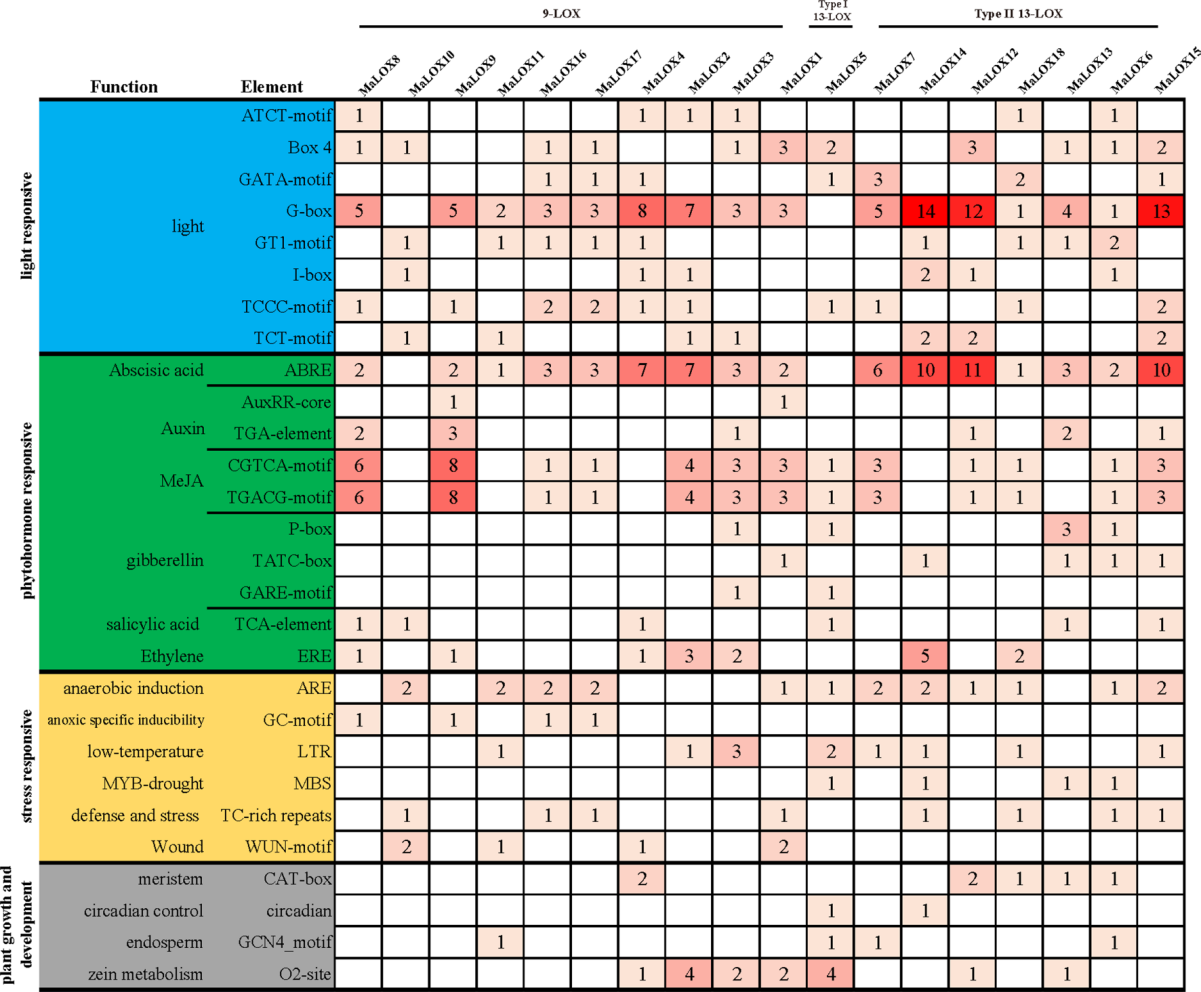
The identified *cis*-acting elements in MaLOX gene family promoters.

In order to further explore the possible expression regulation patterns in the members of the banana LOX gene family, we extracted the promoter sequences of their family members for *cis*-acting element prediction analysis. In total, four categories of *cis*-acting elements were identified, including light responsiveness, phytohormone responsiveness, stress responsiveness, and plant growth and development-related elements (Fig. 6, Supplementary Figures S11, S12). Therefore, it is speculated that the expression of banana *LOXs* may be regulated by multiple factors.

Many light responsive elements are present in the promoters of LOX genes from three banana species, of which the number of G-box elements is the largest. Banana *LOXs* contain at least a *cis*-acting element involved in phytohormone responsiveness classification. Further analysis of the phytohormone responsiveness elements revealed that the number of elements related to abscisic acid was the largest, followed by MeJA. All promoters contain abscisic acid responsiveness elements (ABRE) except *MaLOX5*, *MaLOX10*, *MbLOX9*, *MbLOX10*, *MiLOX1*, and *MiLOX5.* MeJA (TGACG-motif, CGTCA-motif) responsive elements were found in the 30 banana LOX gene promoters (*MaLOX1-3*, *5–9*, *12*, and *15–18*; *MbLOX8-10*, *13–15*, 16, and 18; MiLOX*4*, *5*, *7*, *10*, *12*, *14–16*, and *18*). Furthermore, auxin (TGA-element, AuxRR-core), gibberellin (P-box, TATC-box, and GARE-motif), salicylic acid (TCA-element), and ethylene (ERE) responsive elements are also present on banana *LOX* promoters.

Besides, the promoters also contain several types of stress responsiveness elements, including anaerobic induction (ARE), anoxic specific inducibility (GC-motif), low temperature (LTR), MYB drought-inducibility binding site (MBS), defense and stress (TC-rich repeats), and wound (WUN-motif) responsive elements. Additionally, plant growth and development related *cis*-elements in charge of meristem expression (CAT-box), circadian (circadian), endosperm expression (GCN4_motif), and Zein metabolism (O2-site) regulation were found in the promoter regions of *MaLOXs*, *MbLOXs*, and *MiLOXs*.

### Transcription factor binding site (TFBS) prediction

**Figure 7:**
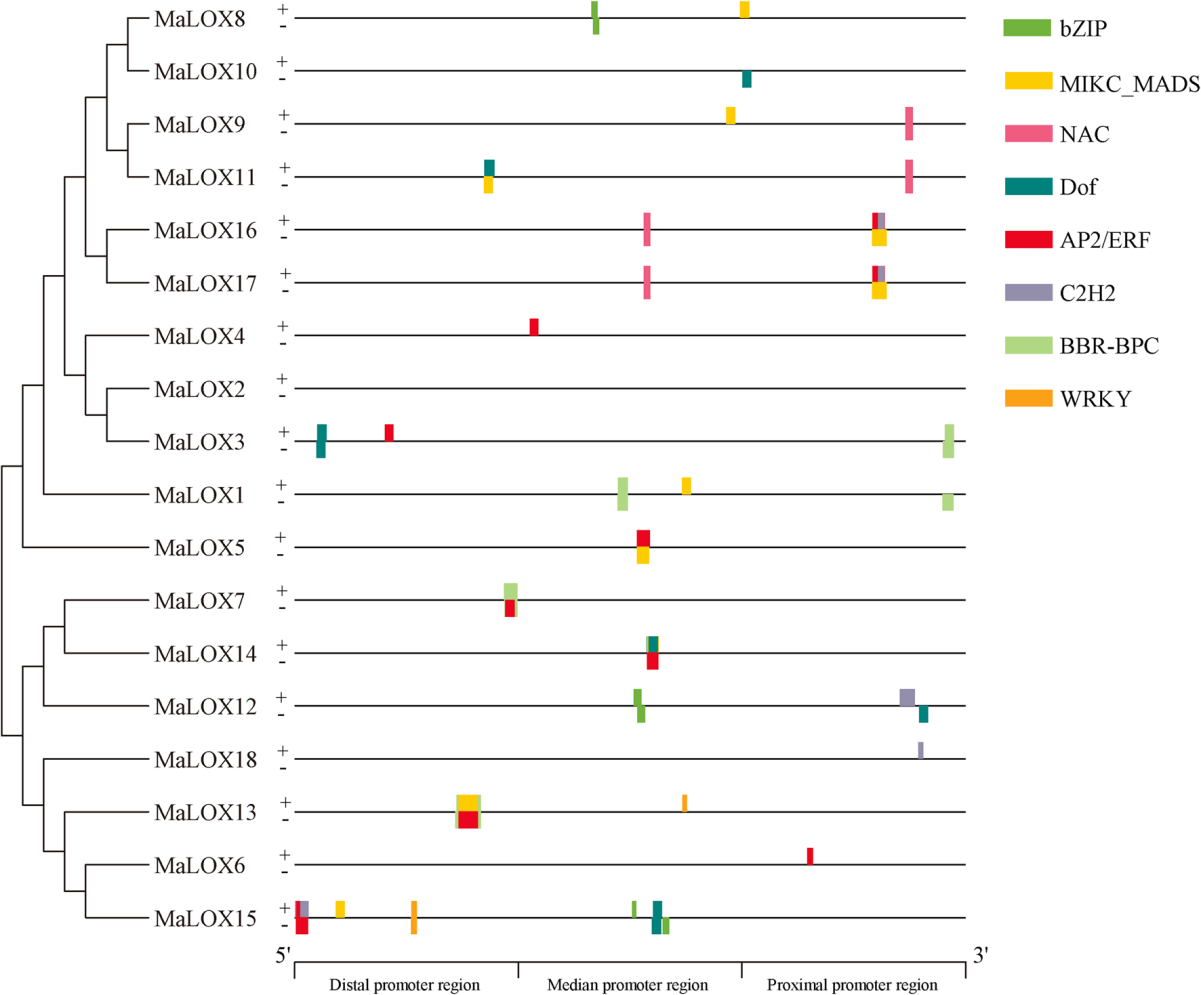
Transcription factor binding sites predicted in the promoters of *MaLOXs.* Boxes of different colors represent different transcription factor families. " + " and "−" represents positive and negative strand, respectively.

To investigate the regulation of transcription factors (TFs) on the expression of banana *LOXs*, transcription factor binding sites (TFBSs) on the promoter were predicted using PlantTFDB online tool. A total of 8 TF families (AP2/ERF, BBR-BPC, bZIP, C2H2, Dof, MIKC_MADS, NAC and WRKY) were identified in the *MaLOX* promoters, which covers 10, 4, 3, 5, 7, 11, 4, and 2 members, respectively (Fig. 7). BBR-BPC family has the largest number of binding sites (51), while WRKY has the least number of binding sites (6). Besides, *MbLOX* and *MiLOX* gene promoters contain six identical TF families, which are AP2/ERF, BBR-BPC, Dof, GATA, MIKC_MADS, and MYB (Supplementary Figure S13A, B). Meanwhile, there are also ARF on the *MbLOX* promoters, and C2H2 and TALE are present in *MiLOX* promoter sequences. In addition, there are certain differences in the TFBS types, number and distribution in the banana *LOX* gene promoters. For instance, 6 types of TF binding sites were found in *MaLOX15*, while *MaLOX2*, *MbLOX14*, and *MiLOX5* were devoid of any TF families. *MaLOX13* has the largest number of TFBS (62), but only 1 TFBS in the promoters of *MaLOX4*, *10*, and *18*, *MbLOX6*, *11*, and *13*, *MiLOX1* and *6*.

### Codon usage bias of MaLOX genes

**Figure 8:**
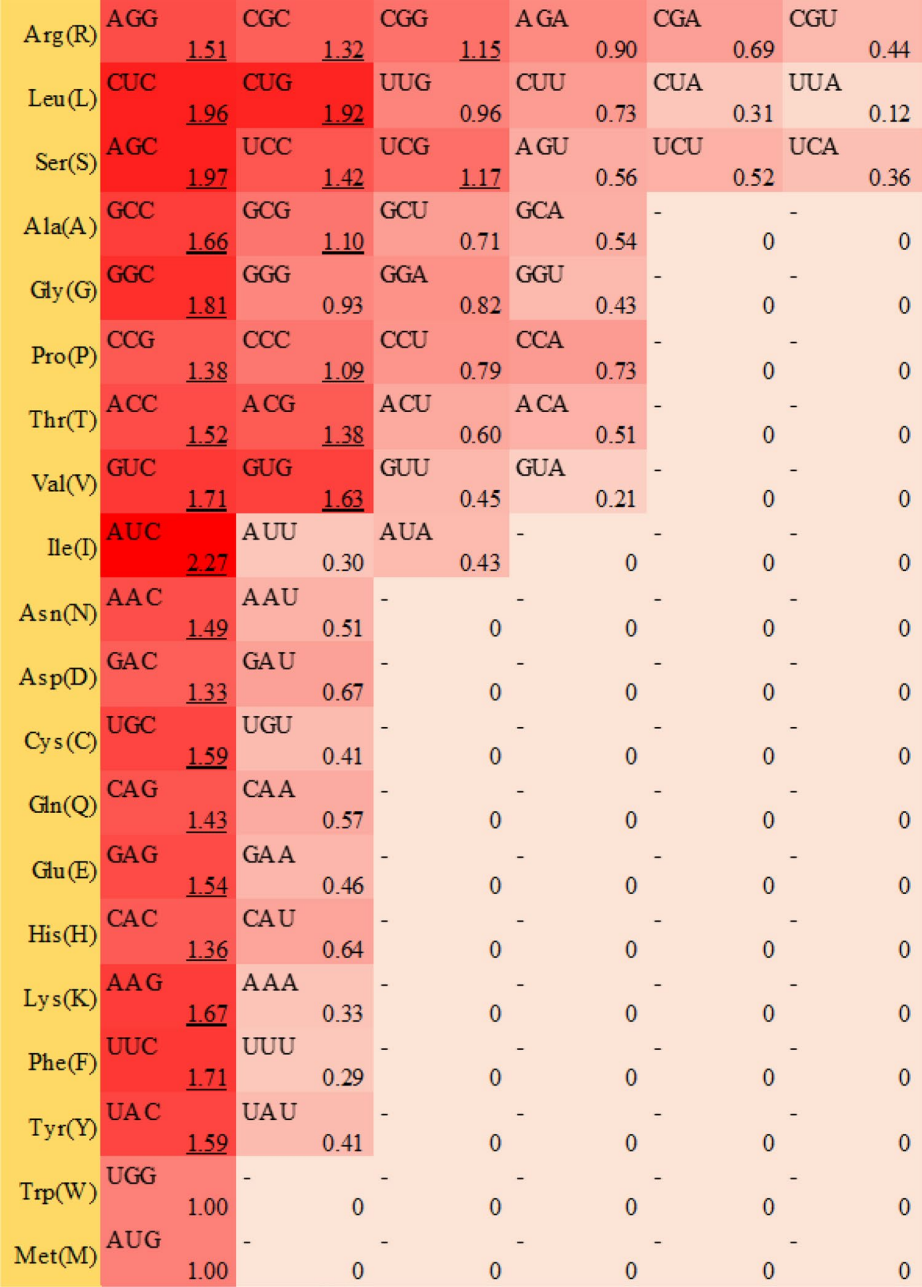
Relative usage of synonymous codons in MaLOX gene family members. The underlined data indicate that the MaLOX genes preferentially to use this codon.

The CodonW software was used to analyze the codon usage bias of the banana *LOX* gene family. Results showed that the effective number of codons (ENC) values of *MaLOXs*, *MbLOXs* and *MiLOXs* are respectively 42.51–56.39, 42.46–54.57, and 42.99–56.70, with an average value of 46.93, 45.81, and 46.94, indicating that the gene expression levels of banana LOX genes were relatively low (Table 3, Supplementary Table S5). The codon adaptation index (CAI) value of *MaLOXs*, *MbLOXs* and *MiLOXs* ranged respectively from 0.18 to 0.26, from 0.18 to 0.26, and from 0.19 to 0.26, with a mean value of 0.23, 0.23, and 0.22, suggesting that the codon bias of banana *LOXs* was weak. With the exception of *MiLOX12*, the average content of C3s and G3s was significantly higher than that of A3s and T3s, and the average content of GC and GC3s was greater than 0.5, which indicated that the banana *LOX* codons generally prefer to use and end with G/C. Relative synonymous codon usage (RSCU) can intuitively reflect the degree to which specified codons deviate from synonymous codons, and RSCU > 1 indicates that the codons are used more frequently than expected. 27 codons showed strong preference for GC-ending codons based on the above criterion in *MaLOXs*, *MbLOXs*, and *MiLOXs*, respectively (Fig. 8, Supplementary Figures S14, S15). Among these, 11 codons end in G and 16 codons end in C.

**Table 3:** Codon preference parameters of *MaLOX* family genes.

Gene name	T3s	C3s	A3s	G3s	CAI	ENC	GC3s	GC
*MaLOX1*	0.14	0.58	0.09	0.45	0.26	42.51	0.81	0.59
*MaLOX2*	0.13	0.54	0.11	0.48	0.26	43.76	0.80	0.60
*MaLOX3*	0.14	0.54	0.12	0.46	0.25	44.44	0.79	0.60
*MaLOX4*	0.14	0.53	0.13	0.45	0.24	45.86	0.78	0.59
*MaLOX5a*	0.23	0.40	0.22	0.37	0.18	55.23	0.62	0.54
*MaLOX5b*	0.18	0.49	0.19	0.40	0.20	50.59	0.70	0.56
*MaLOX6*	0.17	0.46	0.13	0.48	0.20	45.90	0.75	0.60
*MaLOX7*	0.16	0.50	0.13	0.48	0.24	45.68	0.77	0.59
*MaLOX8*	0.15	0.53	0.16	0.41	0.25	46.04	0.75	0.58
*MaLOX9*	0.14	0.54	0.11	0.46	0.25	46.03	0.79	0.59
*MaLOX10*	0.16	0.50	0.18	0.41	0.21	48.06	0.73	0.59
*MaLOX11*	0.14	0.54	0.11	0.46	0.25	45.91	0.79	0.59
*MaLOX12*	0.33	0.29	0.31	0.32	0.19	56.39	0.47	0.48
*MaLOX13*	0.16	0.45	0.14	0.49	0.21	46.10	0.75	0.60
*MaLOX14*	0.22	0.41	0.20	0.43	0.21	53.68	0.66	0.55
*MaLOX15*	0.13	0.51	0.11	0.51	0.22	42.70	0.81	0.61
*MaLOX16*	0.16	0.54	0.15	0.40	0.23	47.11	0.75	0.57
*MaLOX17*	0.14	0.57	0.13	0.41	0.24	42.93	0.78	0.59
*MaLOX18*	0.13	0.52	0.11	0.49	0.24	42.81	0.80	0.61
Average	0.17	0.50	0.15	0.44	0.23	46.93	0.74	0.58

T3s, C3s, A3s, G3s, and GC3s indicate that the third base of the codon is the content of T, C, A, G, and G + C. GC: total GC content in of CDS. CAI: codon adaptation index. ENC: effective number of codons.

### Expression pattern of MaLOX genes under different stresses

**Figure 9:**
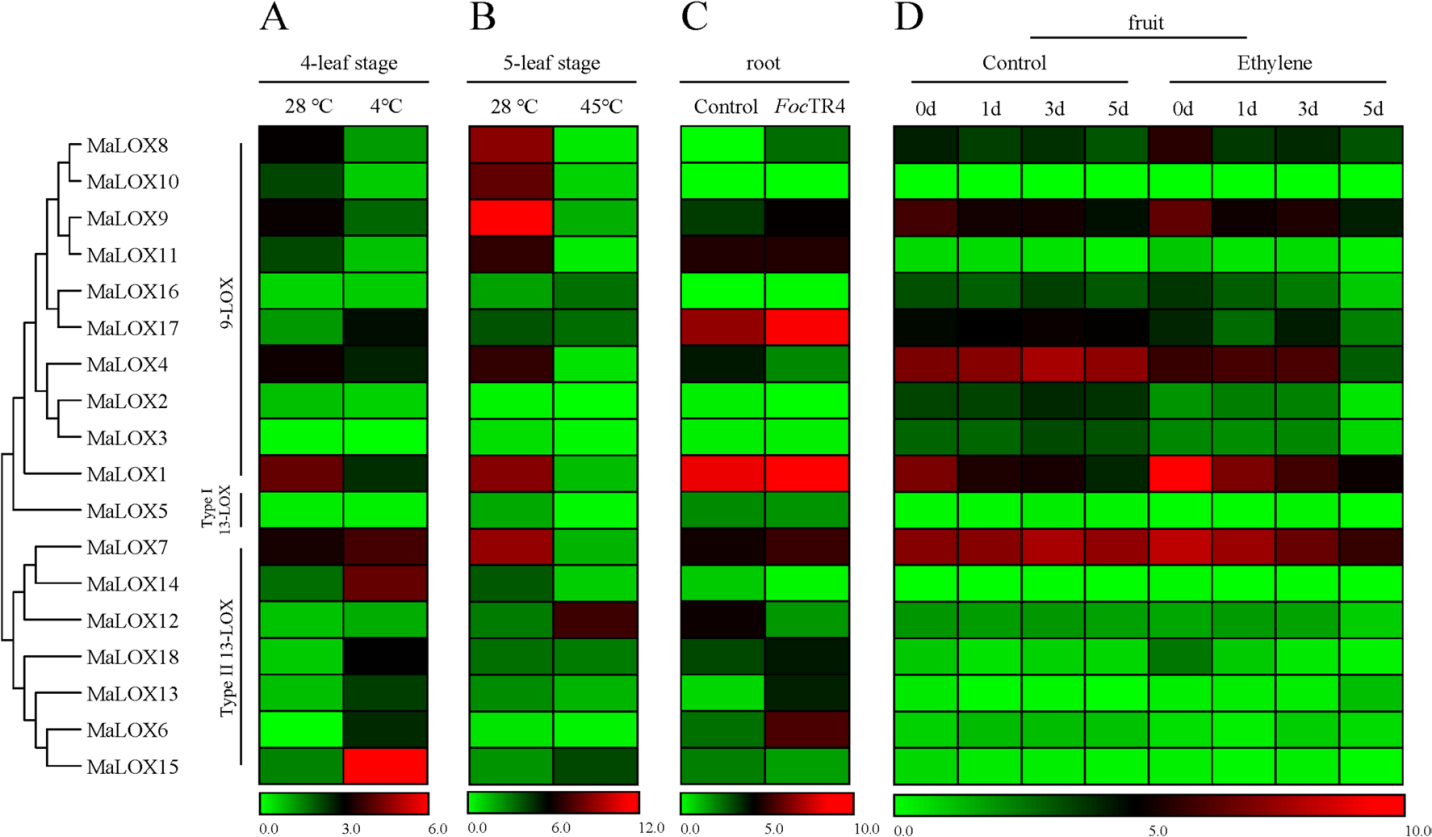
Diagram for the expression of all the MaLOX gene family members. (**A**) Leaf transcriptome data of 4-leaf stage ‘Tianbaojiao’ banana treated with 24 h 4 °C low temperature and 28 ℃ control; (**B**) leaf transcriptome data of 5-leaf stage ‘Tianbaojiao’ banana treated with 3 days 45 °C high temperature and 28 °C control; (**C**) root transcriptome data of *Foc*TR4 treated ‘Tianbaojiao’ banana; (**D**) fruit transcriptome data of ‘Tianbaojiao’ banana at natural ripening and ethylene induced ripening stages.

As shown in Fig. 9, *MaLOXs* showed divergent expression patterns across different tissues (Supplementary Table S6). *MaLOX1* was found to be a highly expressed gene in banana leaves, roots, and fruits. *MaLOX7* was highly expressed in fruits and leaves. The expression of *MaLOX4* in fruits is higher than in leaves and roots. *MaLOX17* was predominantly expressed in the root.

Under low temperature treatment, 6 *MaLOX* members (33.33%) were upregulated and 5 members (27.78%) were downregulated. The expression of most members of the 9-LOX subfamily was inhibited, however, *MaLOX17* was significantly induced (Fig. 9A). Most members of TypeII 13-LOX were upregulated by low temperature, with *MaLOX15* being particularly significant. Under high temperature stress, 3 members (16.67%), including *MaLOX12*, *15*, and *16*, were upregulated, in which *MaLOX12* was significantly induced (Fig. 9B), and 9 members were downregulated. The expression of *MaLOX6, 8*, *9, 13,* and *17* increased greatly under *Foc*TR4 treatment, while the expression of *MaLOX4* and *12* declined (Fig. 9C).

*MaLOXs* expression pattern analysis during natural ripening and ethylene induced ripening was also performed. The expression of the *MaLOX1* was downregulated and *MaLOX8* showed fluctuation change as fruit ripens (Fig. 9D). *MaLOX1*, *7*, *8*, and *18* were upregulated, while *MaLOX2* and *MaLOX4* were downregulated by ethylene at 0 day compared with the control group, but they were downregulated at following timepoints in comparison to the postharvest naturally ripening stage.

### Expression patterns of MaLOX genes under MeJA treatment

**Figure 10:**
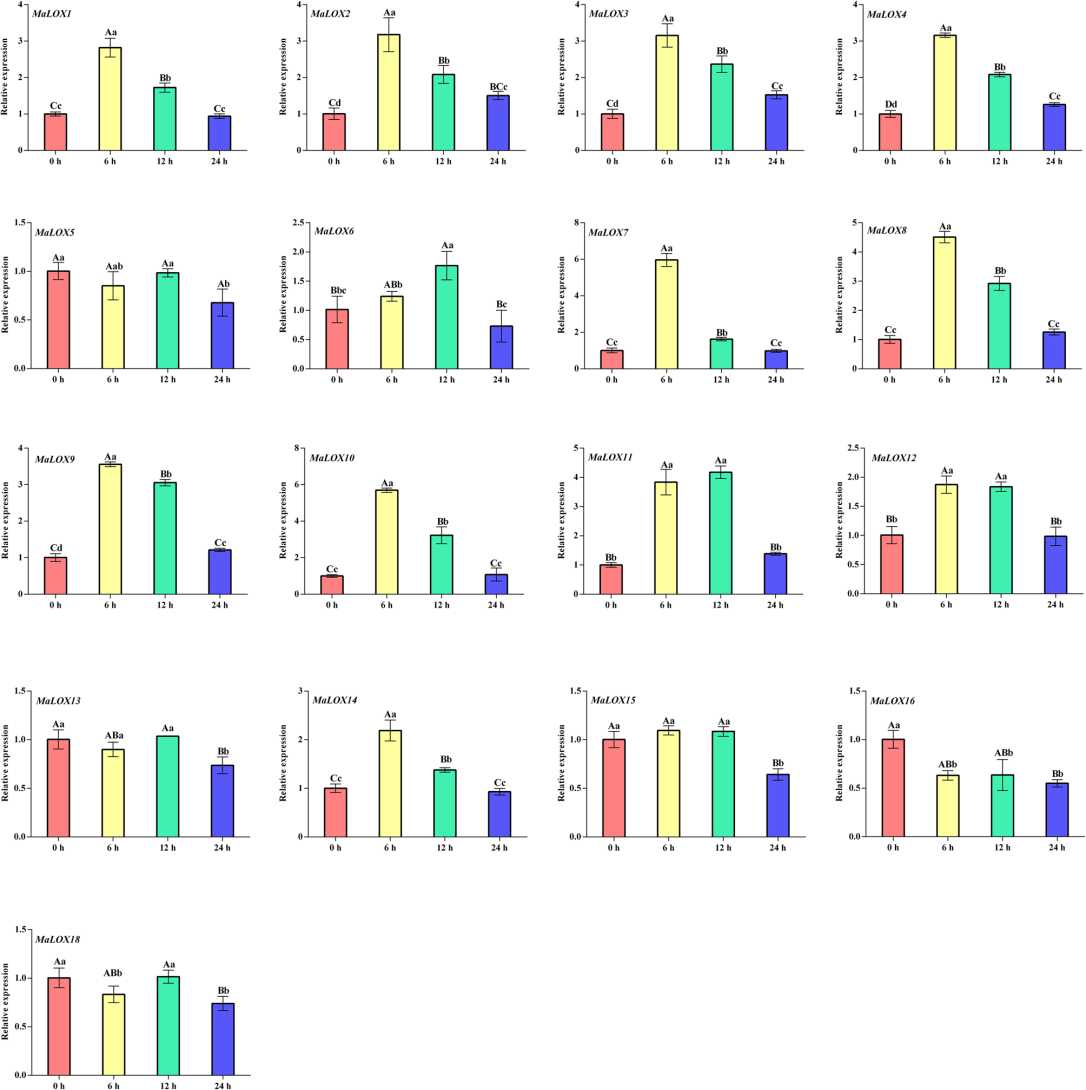
Expression analysis result of MaLOX genes under MeJA treatment. Uppercase and lowercase letters are used to indicate significantly differences at P < 0.01 and 0.05, respectively.

qRT-PCR was performed to determine the responses of the *MaLOXs* to MeJA treatment (Supplementary Table 7). The expression level of *MaLOX17* is too low that its relative expression level was not shown in Fig. 10. The expression of *MaLOX2-4* and *MaLOX9* significantly increased after MeJA treatment, while 5 *MaLOX* members (*MaLOX5*, *13*, *15*, *16*, and *18*) declined significantly. Eight *MaLOX* members (*MaLOX1-4* and *7–10*) were significantly induced by MeJA, and their relative expression peaked at 6 h, then began to decline sharply. *MaLOX6* was significantly upregulated at 12 h post MeJA treatment. *MaLOX12* was dramatically upregulated at 6 h and 12 h and restored to its basal levels during the later periods. The expression level of *MaLOX13*, *15*, and *18* did not change significantly at 6 h and 12 h, but significantly downregulated at 24 h. However, unlike those genes, the expression of *MaLOX14* was significantly induced at 6 h, but afterwards its expression level gradually recovered to the basic level.

### Expression patterns of MaLOX genes under *Foc*TR4 treatment

**Figure 11:**
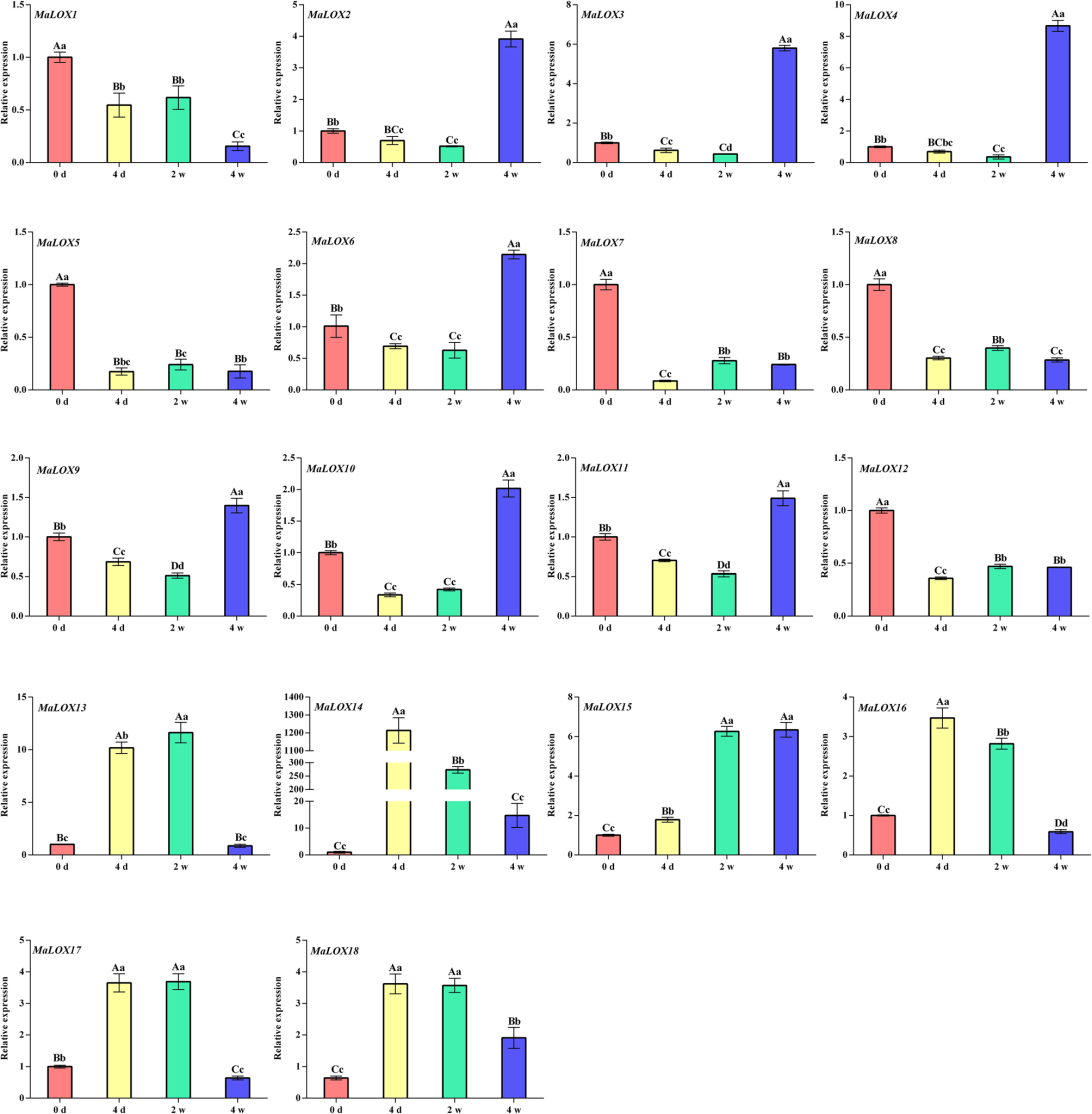
Expression analysis of MaLOX genes after *Foc*TR4 treatment. Uppercase and lowercase letters are used to indicate significantly differences at P < 0.01 and 0.05, respectively.

Gene expression levels of MaLOX genes in response to *Foc*TR4 infection were analyzed using qRT-PCR (Fig. 11, Supplementary Table 8). Within 2 weeks of *Foc*TR4 treatment, the expression level of *MaLOX1*-*12* decreased significantly, among which 4 members (*MaLOX7*, *8*, *10*, and *12*) showed the lowest expression level at 4 days, and *MaLOX3*, *9*, and *11* reached their lowest level of expression at 2 weeks. *Foc*TR4 significantly induced the expression of *MaLOX13-18*, where the expression of *MaLOX13* and *15* gradually increased, and *MaLOX14* and *16* reached their peak at 4 d, and then began to decline. Compared with the 2 weeks, at 4 weeks post *Foc*TR4 treatment, the expression of 7 *MaLOX* members (*MaLOX2-4*, *6*, and *9–10*) was significantly upregulated and reached the maximum, and the expression of 7 members (*MaLOX5*, *8*, *13*, *14*, and *16–18*) was significantly downregulated.

### Weighted gene co-expression network analysis (WGCNA) of MaLOXs

**Figure 12:**
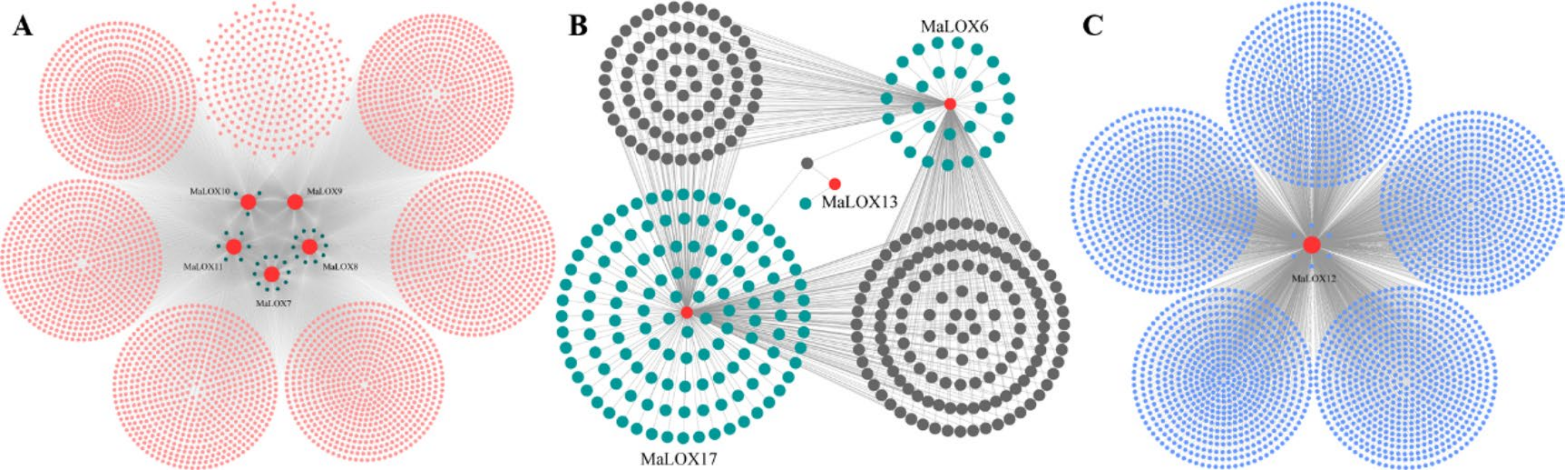
Co-expression network for MaLOX genes. The red nodes indicate MaLOX genes and all other color nodes indicate co-expressed genes with *MaLOXs*. (**A**) Model 1, (**B**) Model 2, and (**C**) Model 3.

**Figure 13:**
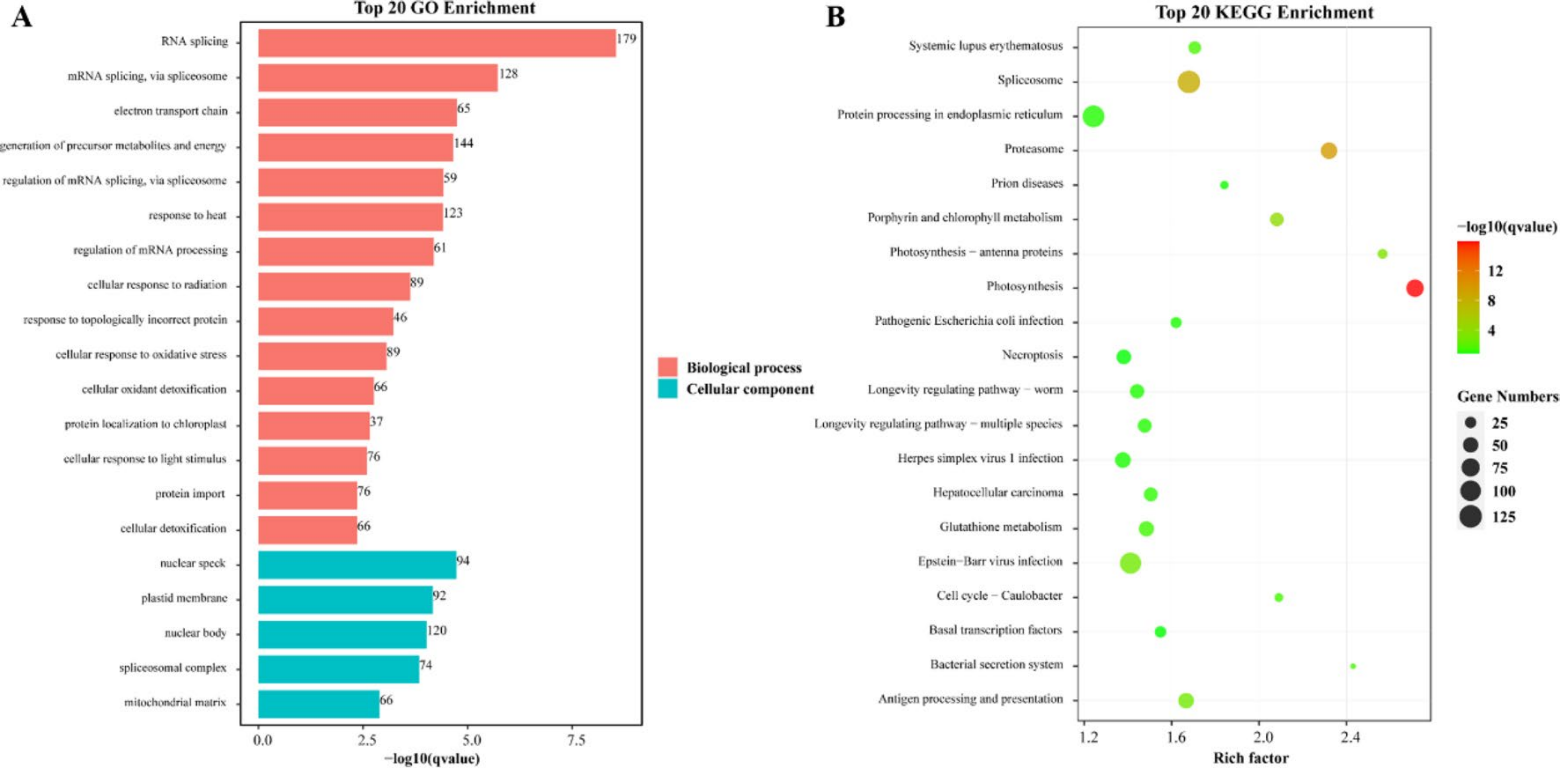
GO and KEGG analysis of the genes in the co-expression network of the MaLOX genes. (**A**) GO functional annotation; (**B**) KEGG pathway enrichment.

To explore the potential interaction and functions between co-expressed genes, WGCNA was applied to construct the co-expression network based on 4 different transcriptome datas, including banana fruit ripening stages, leaves response to high and low temperature, and roots inoculated with *Foc*TR4. We only keep edges with strong connections with weight values ≥ 0.4. A total of 7629 genes were co-expressed with nine *MaLOXs*. Visualization using Cytoscape software, three co-expression networks models, respectively containing 5 (*MaLOX7*-*11*), 3 (*MaLOX6*, *13*, and *17*), and 1 (*MaLOX12*) *MaLOX*, were constructed (Fig. 12). GO enrichment analysis result revealed that, from the aspect of biological process, the *MaLOXs* co-expressed genes were mainly enriched in RNA splicing, mRNA splicing via spliceosome, electron transport chain, generation of precursor metabolites and energy, regulation of mRNA splicing via spliceosome, and response to heat (Fig. 13A); from the aspect of molecular function, nuclear speck, plastid membrane, nuclear body, and spliceosomal complex related co-expressed genes were enriched. According to Kyoto Encyclopedia of Genes and Genomes (KEGG) pathway enrichment results, these *MaLOX* co-expressed genes were found to be enriched in photosynthesis, proteasome, spliceosome, porphyrin and chlorophyll metabolism, and photosynthesis—antenna proteins (Fig. 13B).

## Discussion

### Comprehensive genome-wide identification of LOXs in banana

Lipoxygenase is a crucial restriction enzyme in the LOX pathway, which catalyzes the fatty acid metabolism of plant, actively participates in growth and development, and resists extreme external environmental conditions^38^. In this study, we identified 18 MaLOX, 11 MbLOX, and 12 MiLOX genes from *M. acuminata*, *M. balbisiana*, and *M. itinerans* genome, respectively. *MaLOXs* have more members than *Arabidopsis* (6), rice (14), and tomato (14), but are the same as grapes (18)^39^ and melon (18)^40^. Besides, the number of LOX in banana from most to least is *MaLOXs* > *MiLOXs* > *MbLOXs*, which is consistent with their genome size (501.5 MB for *M. acuminata*, 430 MB for *M. balbisiana* and 462.1 MB for *M. itinerans*).

Phylogenetic analysis showed that the banana LOXs family could be further divided into three subfamilies, including 9-LOX, Type I 13-LOX, and Type II 13-LOX, which was consistent with the results of poplar^9^ and tea plant^10^. The sequence similarity among Type I LOX members ranged from 26.90 to 98.90%. Type II 13-LOX members contained chloroplast transit peptides except MiLOX6 and MiLOX18, and their sequence similarity ranged from 44.90 to 92.60%. Our results are not completely consistent with classification method of Shibata et al.^41^, who put forward that Type I LOX genes exhibit high sequence similarity (more than 75%) and lack of chloroplast transit peptide, while Type II LOX genes show moderate overall sequence similarity (up to 35%) and exist chloroplast transit peptide. But our result was consistent with the melon LOXs^40^, which may be related to the diversity of the evolution process of the LOX genes. The prediction of subcellular localization showed that MaLOX18, MbLOX18 and MiLOX18 were localized in the cytoplasm, while other Type II 13-LOX members were all localized in the chloroplast. This may be due to the poor conservation of the amino acid sequence of the chloroplast transit peptide of banana LOX18^42,43^. The members of this subfamily have similar gene structure and conserved motifs, indicating that the gene function of banana LOX members from the same subfamily showed certain degree of conservativeness. Our study found that codon bias of banana *LOXs* was weak, preferring to use and end with G/C, which is consistent with the codon preference characteristics of monocotyledon plants^44^ and banana genome^45^. Thus, it was hypnotized that in order to cope with environmental pressures, different banana species have formed unique codon usage bias during evolution.

### LOXs may play special roles in banana evolution

Gene duplication is a major factor responsible for the amplification in family gene numbers, in which whole genome duplication (WGD) is considered to be an important driving force for expansion and an important source of gene function diversification^4^. There are three pairs of segmental duplication genes and three tandem duplication gene clusters in the poplar LOX family genes^9^. Five tandem repeat pairs were observed in tomato LOX family, and no segmental duplicate pairs^8^. In this study, the four pairs of segmental duplication genes and two pairs of tandem repeat genes were found in *MaLOX* gene family, accounting for 27.78% (4/18) and 22.22% (5/18), respectively. Banana is speculated to undergo three whole genome duplication events during the evolution, which were α, β, and γ events, respectively^46^. The duplication events of the MaLOX genes were supposed to originate from 0.09 to 110.20 Mya, of which *MaLOX1/MaLOX16* dated the duplication event at 110.20 Mya, corresponding to the γ event. The tandem repeat event of *MaLOX8/MaLOX9* occurred at 62.65 Mya, corresponding to the α or β event. It was suspected that the whole genome, segmental, and tandem duplication together contributed together to the expansion of MaLOX gene family. Moreover, MbLOX gene family also contains three pairs of segmental duplication genes and three pairs of tandem repeat genes, accounting for 27.27% (3/11) and 36.36% (4/11), which indicates that tandem duplications and segmental duplications together play a role in the expansion of MbLOX gene family.

The mechanism of gene and genome evolution can be understood through a comparative analysis of relatively close between-species genome. This study has found that there are a high conservation level and have a close homology relationship among MaLOX, MbLOX, and MiLOX genes. The ancestor of *M. acuminata and M. itinerans* diverged with *M. balbisiana* ancestor about 8.3 Mya, and *M. acuminata* and *M. itinerans* diverged about 5.8 Mya, while the divergence time was about 5.4 Mya for the *M. acuminata* and *M. balbisiana*^47,48^. We found that 5 of 14, 8 of 12, and 6 of 9 orthologous gene pairs appeared after the divergence of *M. acuminata*/*M. balbisiana*, *M. acuminata*/*M. itinerans*, and *M. balbisiana*/*M. itinerans*. *M. acuminata* and *M. balbisiana* shared less orthologous gene pairs with *M. balbisiana*, which may be explained that *M. balbisiana* genome exhibited less expansion and more contraction of gene families after divergence and *M. acuminata* and *M. itinerans* have relatively higher similarity^47,48^.

Besides, evolutionary selection pressure analysis of banana LOX duplication genes showed that *MaLOX16/MaLOX17* experienced strong positive selection pressure, indicating that functional differentiation occurred. And other duplication genes were subject to purification selection pressure and limited functional differentiation^5^.

### Functional prediction of *MaLOXs*

The *cis*-acting elements of the promoter combine with specific transcription factors to form transcription initiation complex and initiate gene specific expression^49^. Four types of *cis*-regulatory elements were identified at the banana *LOX* promoters, including light, phytohormone, stress, growth and development-related, which is consistent with the report about the functional diversity of *LOX* genes^50^. Besides, a variety of kinds of TFBSs were found in the banana *LOX* promoters. Recent research demonstrated that TFs play an important role in banana growth and adversity stress^51–53^, and it is further speculated that banana *LOX* expression is regulated by many TFs.

Lipoxygenase is a kind of oxygenase widely distributed in various organs of plants, and its expression levels in different parts and developmental stages of plants differed, which are closely related to physiological processes such as plant growth, development, maturity, and senescence^10,37^. In this study, each member of the *MaLOX* family was expressed in at least one organ. *MaLOX1* was highly expressed in leaf, root, and fruit, which suggests that the function of *MaLOX1* may be diverse. The expression of *MaLOX4* in fruit is higher than in leaf and root, and *MaLOX7* is highly expressed in fruit and leaf, which means that the functions of different *MaLOXs* members varied in different organs.

Low temperature can inhibit the transcriptional level of *LOX* in banana fruit, reduce the banana volatiles, and the inhibition effect is more obvious as the temperature decreases^25^. Under high temperature, there is an overall decrease in the amount of LOX proteins in banana peel^26,27^. Li et al.^28^ found that the high expression of *LOX* was related to higher *Foc*TR4 resistance of resistant mutant. *LOX1.1–3* and *LOX2.3* were significantly induced in resistant variety (*Musa yunnanensis*) during early infection with *Foc*TR4^54^. In this study, the analysis of transcriptome data under low temperature, high temperature, and *Foc*TR4 treatment revealed that the expression patterns of *MaLOXs* under different stresses differed. *MaLOX8*, *9*, and 13 responded significantly to the above three stresses. The expressions of *MaLOX1*, *8*, *10*, *11*, *14*, and *15* were regulated by high and low temperature; *MaLOX6* and *17* were induced by low temperature and *Foc*TR4; *MaLOX4* and *MaLOX12* responded to high temperature and *Foc*TR4. *MaLOX7* and *16* were differentially expressed at high temperature and *MaLOX18* was only induced by low temperature. In addition, this study also found that in the early stage of *Foc*TR4 infection, each member of *MaLOXs* responded to varying degrees.

WGCNA is an effective way to identify clusters of highly correlated genes and can better preserve the characteristics of biological networks and reflect the relationship among functions and different biological processes^55,56^. Most of the adjacent genes of *MaLOXs* in their co-expression network were related to RNA splicing, generation of precursor metabolites and energy, heat stress, photosynthesis, and proteasome. Besides, the promoter regions of these differentially expressed genes contain a large number of stress-related *cis*-acting elements and TFBSs. These results indicated that *MaLOXs* are widely involved in banana growth and development and various stress responses.

*LOX* regulates the processes of plant ripening and senescence by participating in the synthesis of ethylene or catalyzing polyunsaturated fatty acids to generate superoxide radicals and destroying cell membrane structure^40,57^. And the roles of *LOX* in fruit ripening and flavor formation have been confirmed in tomato^8^, apple^58^, peach^12^ and kiwi^59^. Our study found that *MaLOX1* was downregulated during fruit ripening and 6 members (*MaLOX1*, *2*, *4*, *7*, *8*, and *18*) were found to be ethylene responsive. It was reported that under ethylene and high-temperature treatment, the content of LOXA, LOX4, and LOX5 (corresponding to MaLOX4, MaLOX8, and MaLOX1 in this study, respectively) decreased in banana fruit peel^26^. During banana fruit ripening, the expression of *MaLOX* (or named as *BanLOX*) decreased^60^. After ethylene treatment, however, it was upregulated in the pulp while it did not change significantly in the peel^60^, which is similar to the results of this study. Moreover, *MaLOX1*, *4*, and *7* were predominantly and specifically expressed during fruit ripening and were regulated by ethylene. Therefore, we speculated that these LOX genes may be the candidate genes involved in banana fruit ripening and flavor formation.

MeJA/JA, as a signal molecule that affects biological and abiotic reactions in plants, plays an important role in dealing with various external stresses. It was found that the application of exogenous MeJA can induce endogenous JA biosynthesis in plants^61^, and JA biosynthesis mainly depends on the substrate and expression of the genes at the critical steps of the synthesis pathway, such as *LOX*, *AOC*, *AOS*, and *OPR*^62^. In this study, with the exception of *MaLOX11*, *16,* and *17*, most 9-LOX subfamily *MaLOX* genes were upregulated and reached the maximum expression level at 6 h. And the expression trend of *MaLOX14*, belonging to Type II 13-LOX subfamily, also showed similar expression pattern. The expression of *MaLOX16* is suppressed by MeJA, while *MaLOX13*, *15*, *18* were significantly upregulated at 24 h. We also found that most MeJA responsive *MaLOXs* contain MeJA-responsive elements in their promoters. The expression of poplar^9^, *Panax ginseng*^63^, pepper^64^, and tomato^8^ LOX genes were found to be regulated by MeJA to some extent, which is consistent with our results. In addition, external application of MeJA can induce the expression of *MaLOX1* and *MaLOX2*, enhance the content of endogenous JA, and alleviate banana chilling injury partially^62^. The above results indicate that MeJA can cause the up-regulation of LOX genes, which can increase the content of endogenous JA, thus improve the stress resistance of plants.

## Conclusions

In this study, 18, 11, and 12 family members were respectively identified from *M. acuminata*, *M. balbisiana*, and *M. itinerans* genome, which encoded proteins with conserved domains and mainly located in the cytoplasm or chloroplast. The condon usage in banana *LOX* family members prefer to use and end with G/C. Four segmental duplications and 2 tandem duplications as well as 3 segmental duplications and 3 tandem duplications occurred respectively during *M. acuminata* and *M. balbisiana* evolution. Banana LOXs can be divided into three subfamilies, including 9-LOX, Type I 13-LOX, and Type II 13-LOX, and the sequence characteristics between each subfamily members are conservative. The expression of *MaLOXs* showed certain tissue specificity, and showed different response patterns to MeJA, high temperature, low temperature, and *Foc*TR4 treatments. Moreover, the potential function analysis of the protomer region and the co-expression network of *MaLOXs* was constructed using WGCNA indicated that *MaLOXs* might participate in the growth and development and various stress responses in banana. Our present study can extend the knowledge of banana LOX gene family and provide basis for future exploration of their functions.

## Supplementary Information

Supplementary Figures.

Supplementary Table S1.

Supplementary Table S2.

Supplementary Table S3.

Supplementary Table S4.

Supplementary Table S5.

Supplementary Table S6.

Supplementary Table S7.

Supplementary Table S8.
