# Supplementary material for: Genome-wide identification and expression pattern analysis of lipoxygenase gene family in banana
Source: Sci Rep. 2021 May 11;11:9948. doi: 10.1038/s41598-021-89211-6 (PMC8113564; doi:10.1038/s41598-021-89211-6)
Supplement: Supplementary file 1 — Supplementary Figures. [file 41598_2021_89211_MOESM1_ESM.pdf]

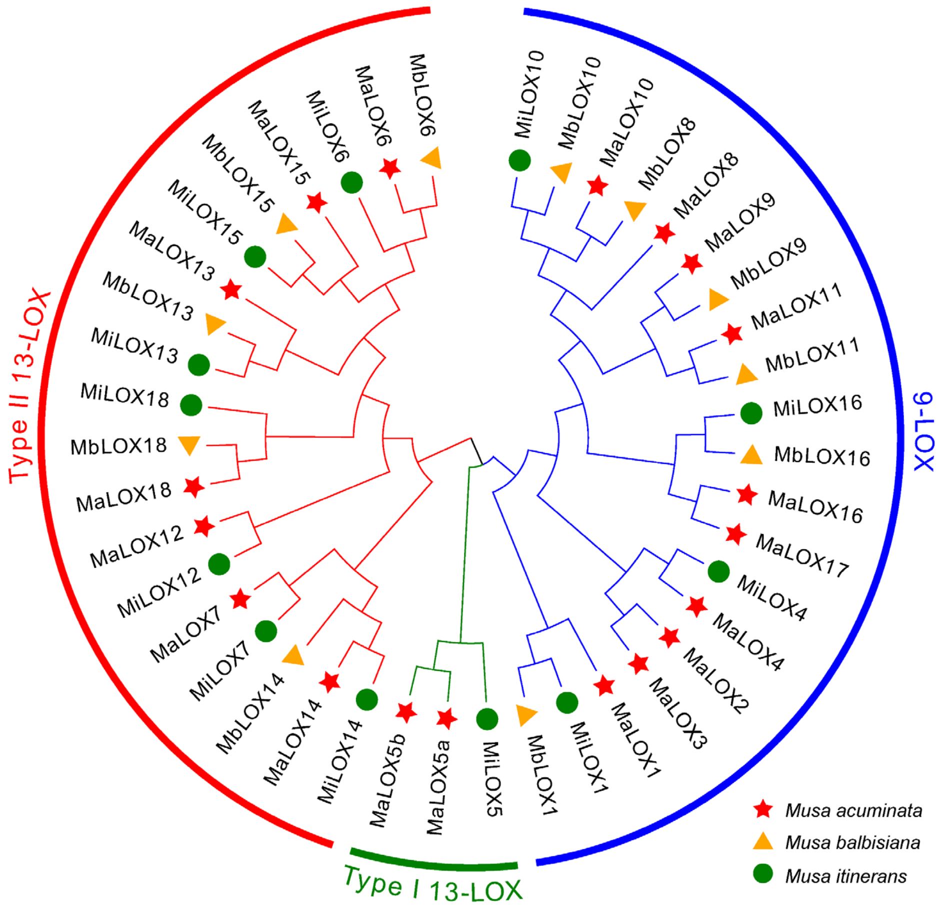

Figure S1. Phylogenetic tree of banana LOX genes family.

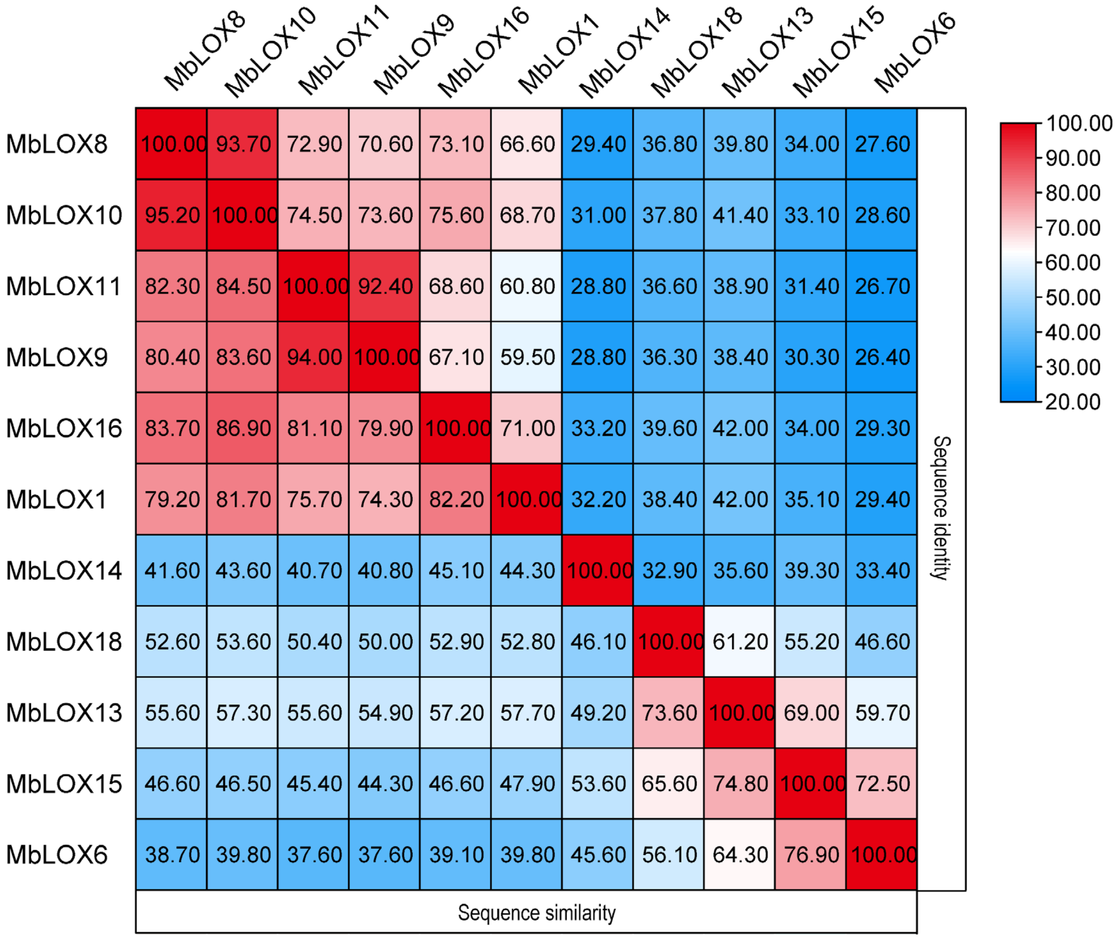

Figure S2. Sequence identities and similarities (%) among the *MbLOXs*.

	MiLOX15	MiLOX6	MiLOX13	MiLOX18	MiLOX12	MiLOX7	MiLOX14	MiLOX5	MiLOX1	MiLOX4	MiLOX16	MiLOX10
MiLOX15	100.00	83.90	84.70	64.20	47.60	41.70	41.40	36.20	40.90	39.40	35.30	38.90
MiLOX6	88.90	100.00	81.90	62.80	46.90	40.20	40.30	38.20	43.30	41.40	38.40	40.80
MiLOX13	91.00	88.20	100.00	63.00	48.80	42.20	40.90	35.80	40.70	39.30	36.20	38.70
MiLOX18	76.20	73.80	75.60	100.00	47.00	39.30	39.10	34.00	38.40	38.00	35.00	36.30
MiLOX12	62.40	61.00	64.20	62.20	100.00	44.50	45.50	33.90	38.50	37.40	35.60	37.00
MiLOX7	55.50	53.80	56.10	53.70	61.10	100.00	64.60	38.30	35.30	37.90	35.40	36.10
MiLOX14	55.10	53.70	54.20	53.70	62.50	77.60	100.00	36.20	36.30	37.50	35.30	37.60
MiLOX5	51.20	53.10	49.80	48.60	50.30	53.50	50.10	100.00	47.90	46.40	43.00	47.50
MiLOX1	56.80	59.50	55.70	53.20	54.50	48.30	50.00	64.10	100.00	65.20	60.80	66.30
MiLOX4	54.20	56.50	53.20	52.10	51.40	50.80	49.90	62.40	78.00	100.00	66.30	73.00
MiLOX16	49.00	51.60	49.00	47.50	48.60	47.30	47.50	58.50	70.50	76.20	100.00	68.20
MiLOX10	54.40	56.80	53.20	52.40	52.70	51.20	51.80	64.20	79.10	85.30	78.00	100.00

Sequence identity

Sequence similarity

100.00
90.00
80.00
70.00
60.00
50.00
40.00
30.00

Figure S3. Sequence identities and similarities (%) among the *MiLOXs*.

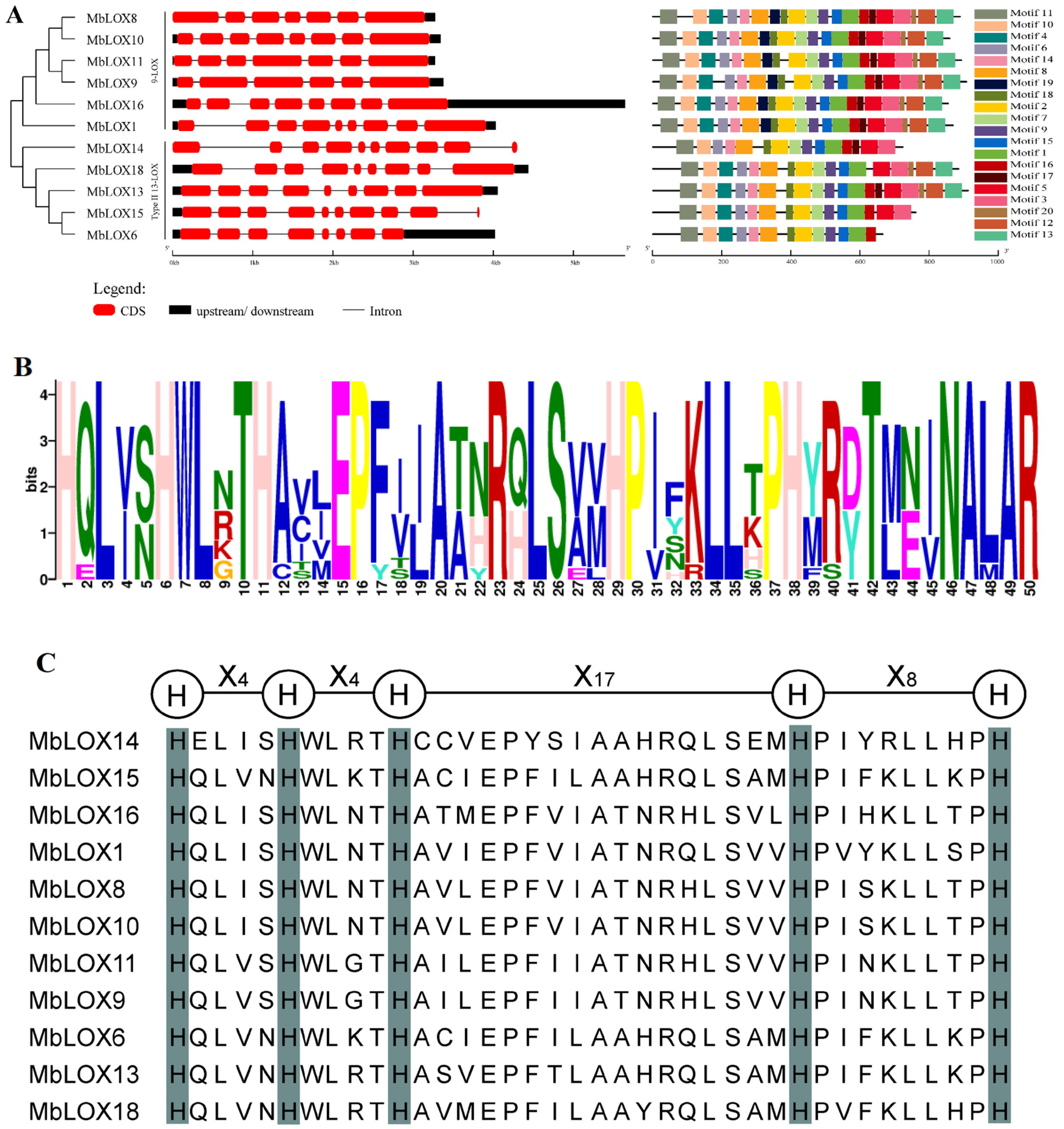

Figure S4. Gene structures and conserved motifs (A), Motif 1 sequence (B) and the 38 conserved residues of MbLOX proteins of *MbLOXs* or their encoded proteins. the dark color in C shows highly conserved histidine (His).

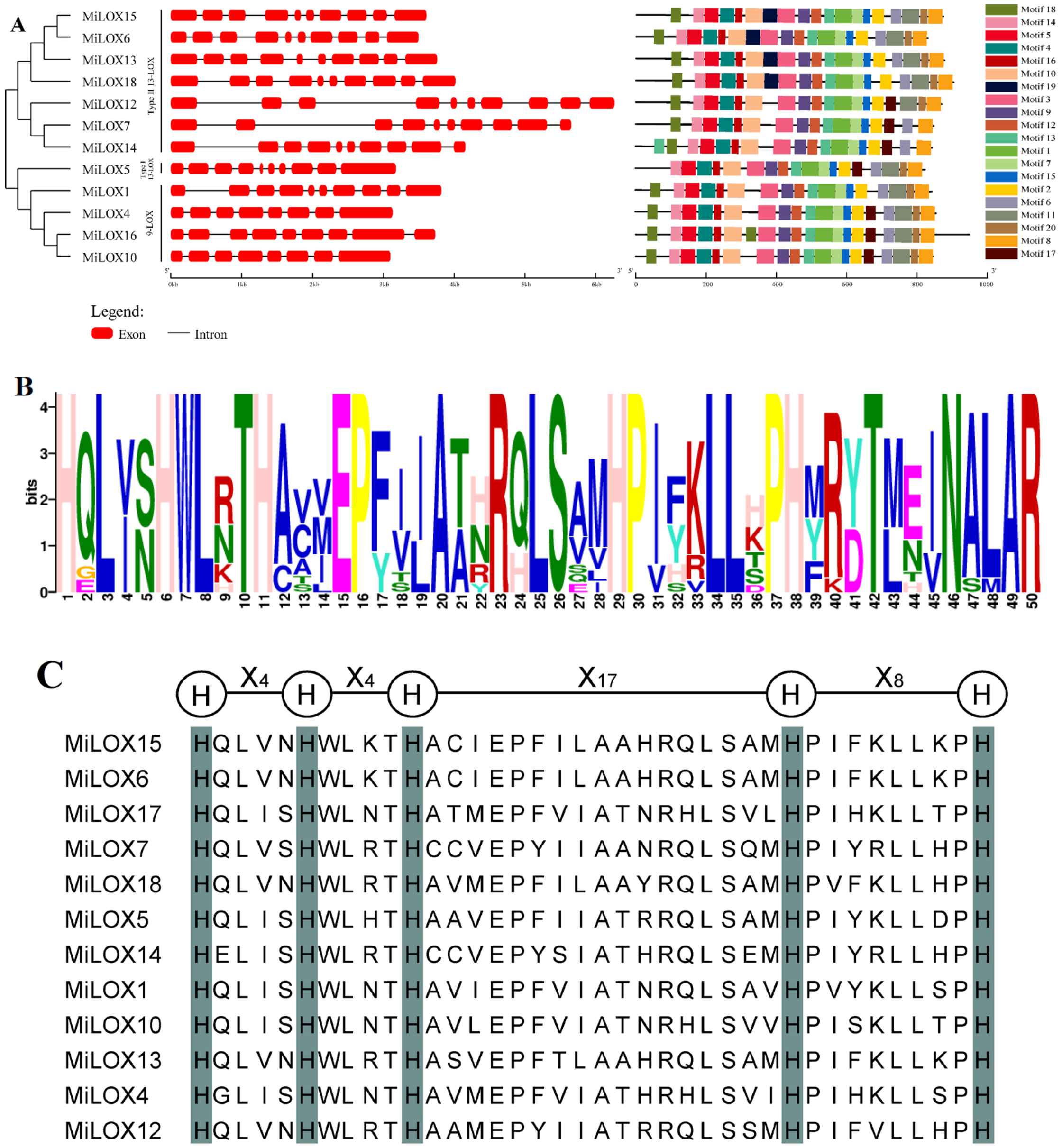

Figure S5. Gene structures and conserved motifs (A), Motif 1 sequence (B) and the 38 conserved residues of MiLOX proteins of *MiLOXs* or their encoded proteins. the dark color in C shows highly conserved histidine (His).

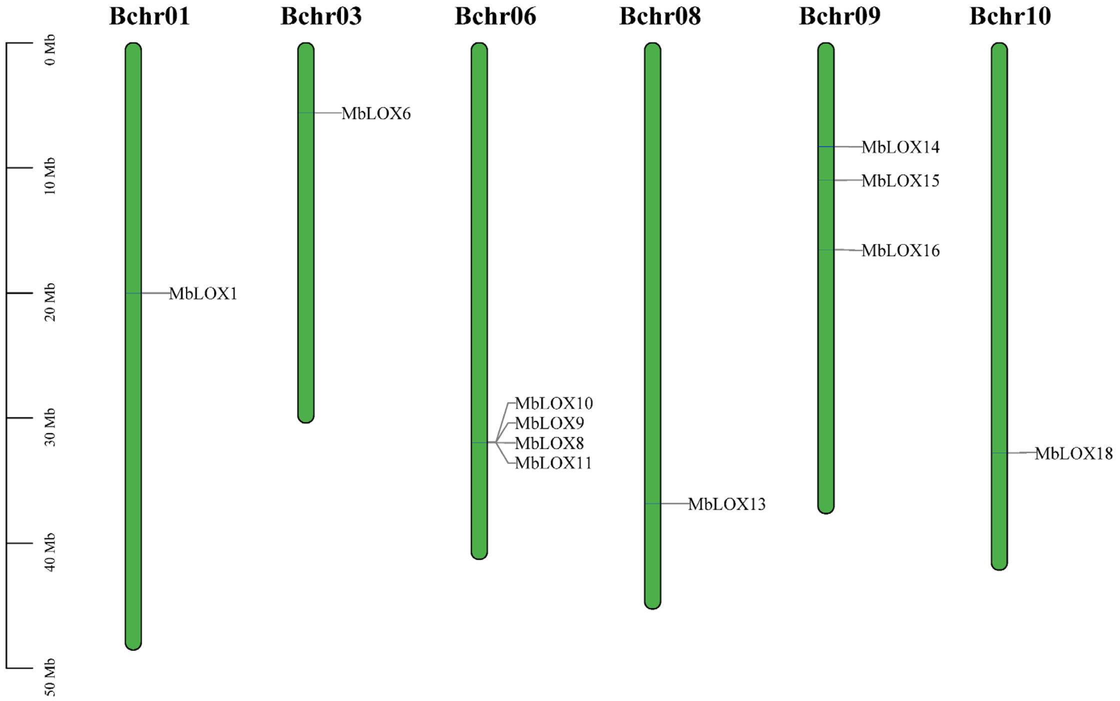

Figure S6. Chromosome localization of MbLOX genes.

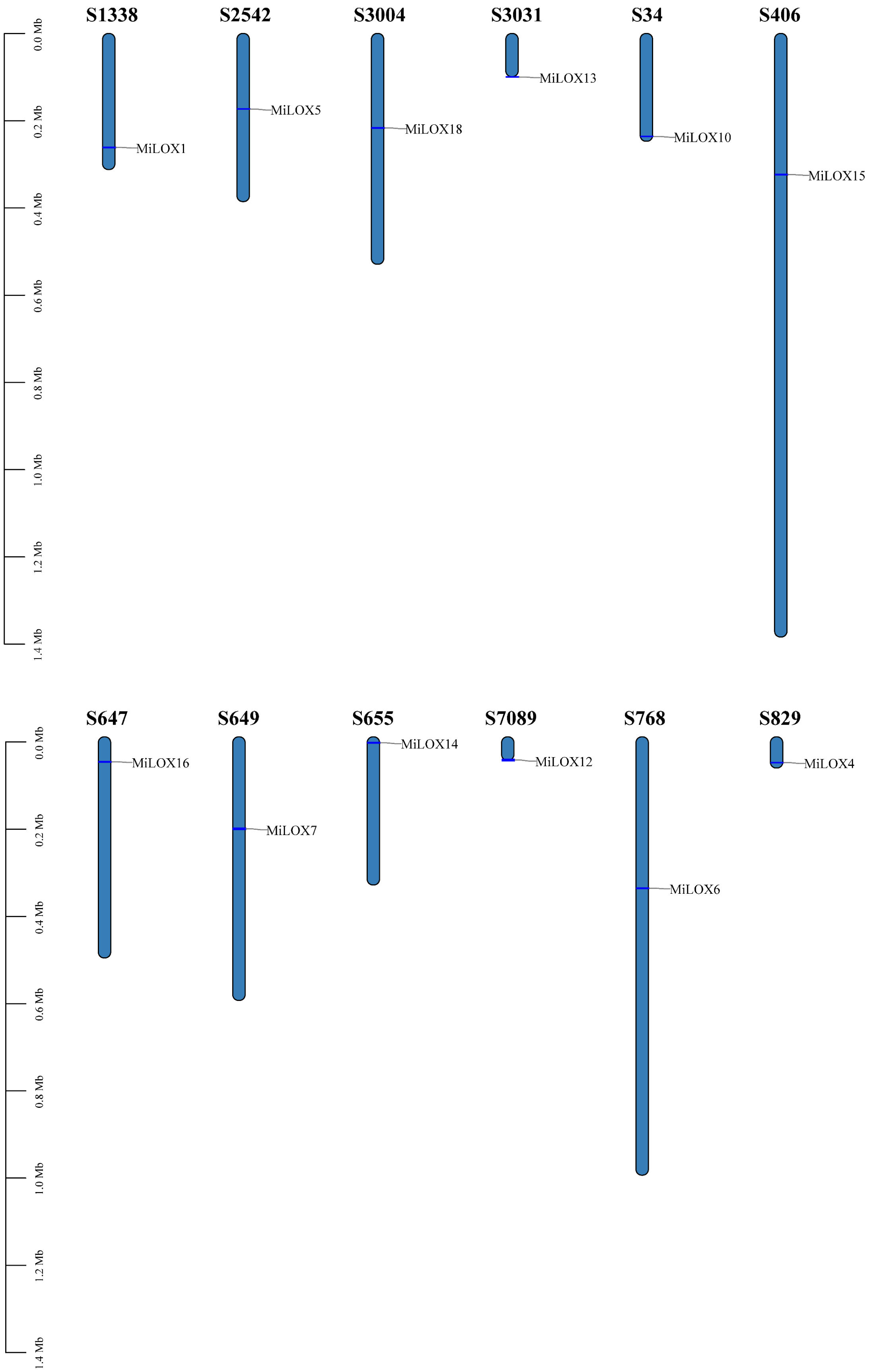

Figure S7. Chromosome localization of MiLOX genes.

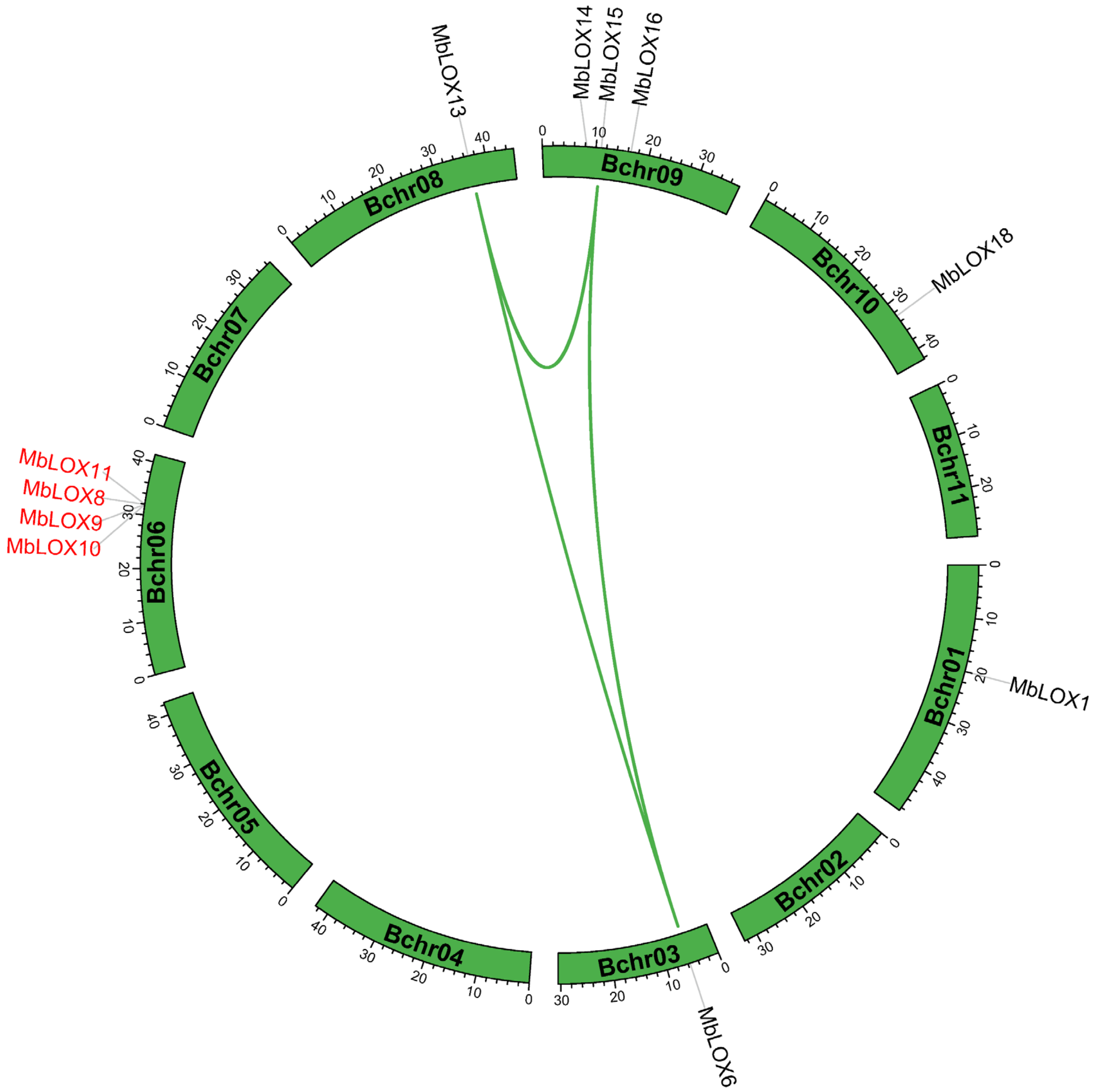

Figure S8. Collinear distribution of MbLOX genes. The green line indicates the collinearity between the *MbLOXs*, and the gene names in red are tandem replication genes.

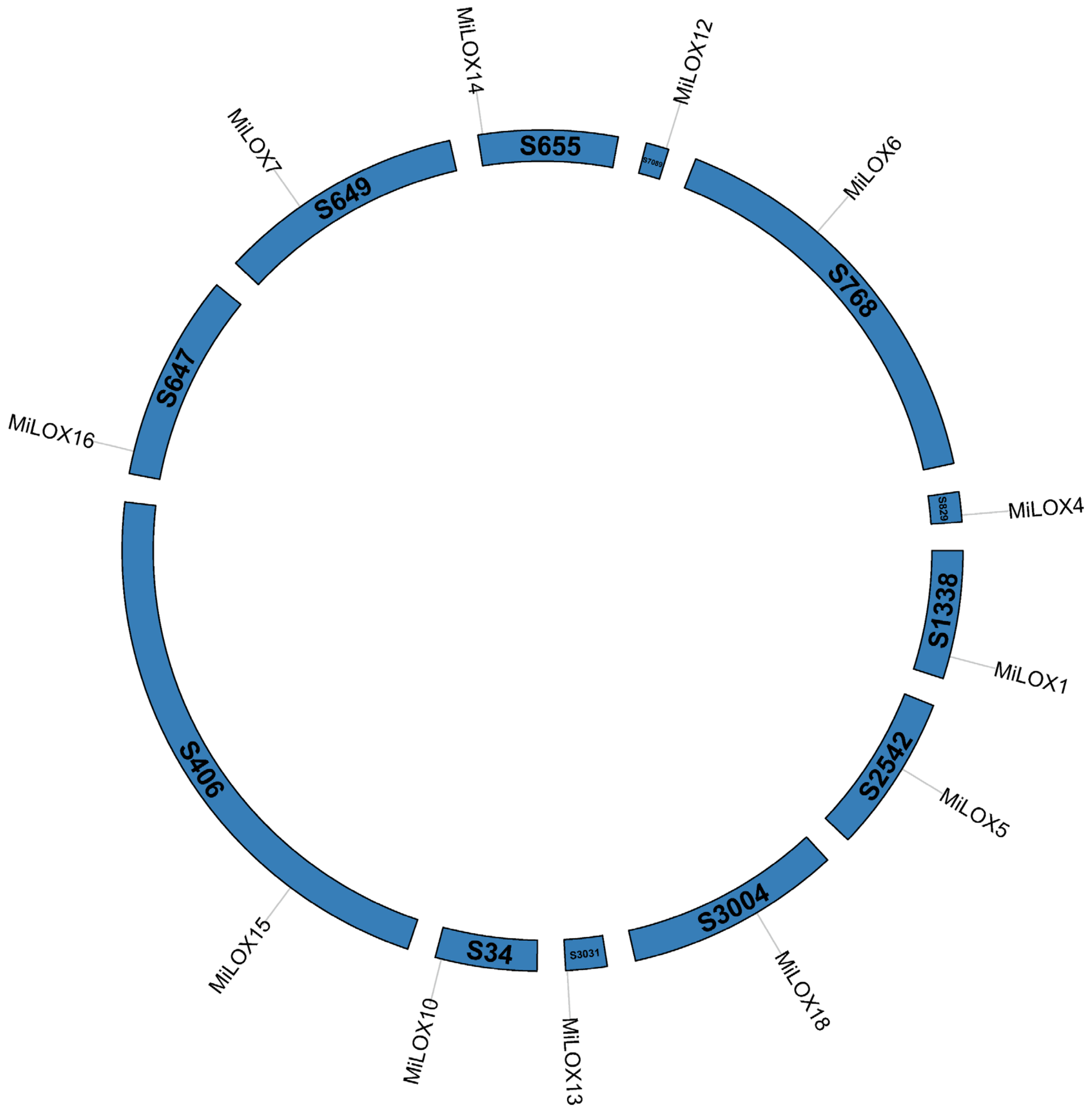

Figure S9. Collinear distribution of MiLOX genes.

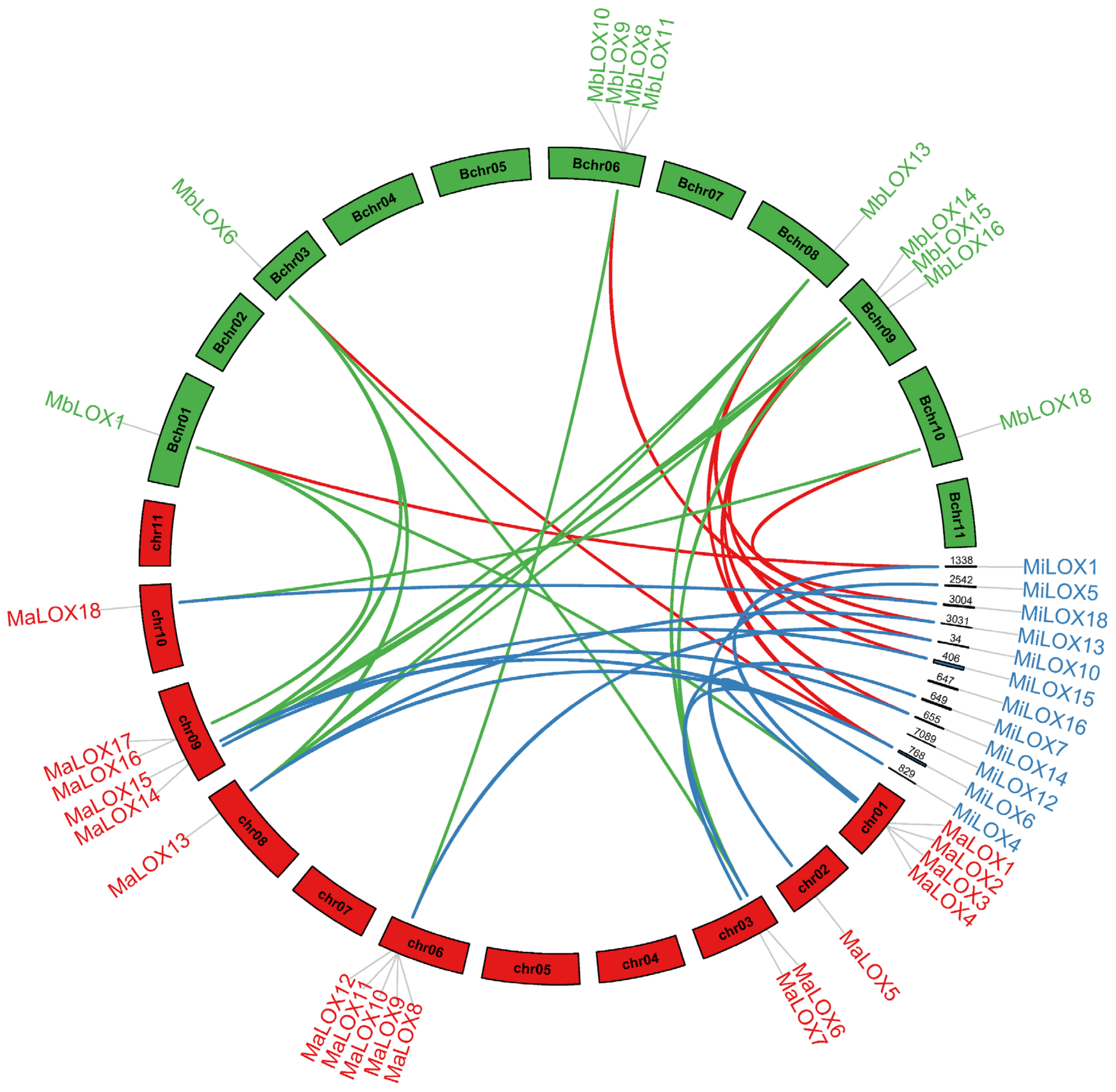

Figure S10. Orthologous among *M. acuminata, M. balbisiana* and *M. itinerans*.

Category	Function	Element	MbLOX8	MbLOX10	MbLOX11	MbLOX9	MbLOX16	MbLOX1	MbLOX14	MbLOX18	MbLOX15	MbLOX6	MbLOX13
light responsive	light	G-Box	2		6		2	2	2	4	7	4	5
		Box 4	1	1		1	3	3		1	1	4	1
		TCCC-motif		1			2				1	2	
		GATA-motif		2			1			1	2	1	
		AE-box					2		6				
		GT1-motif					1		1	1		2	1
		Sp1							1	3			1
		I-box	1	1	1	1			1			2	
phytohormone responsive	Abscisic acid	ABRE	2		5		2	1	1	5	5	2	4
	Auxin	AuxRR-core						1	3				
		TGA-element		3		2	1		1		1		3
	MeJA	CGTCA-motif	1	1		2	1		1	2	2		2
		TGACG-motif	1	1		2	1		1	2	2		2
	gibberellin	P-box								2		1	3
		GARE-motif											
		TATC-box							1				1
	salicylic acid	TCA-element		1		1						1	
	Ethylene	ERE		1		1			9				1
stress responsive	anaerobic induction	ARE	1	1	1			3	1	2	1	1	
	anoxic specific inducibility	GC-motif								1			1
	low-temperature	LTR	1		1				1	1	1		
	MYB-drought	MBS							1			1	1
	defense and stress	TC-rich repeats				1		1			1		
	Wound	WUN-motif			1					1		1	
plant growth and development	meristem	CAT-box								1			1
	circadian control	circadian						2	1				
	endosperm	GCN4_motif				1						1	
	zein metabolism	O2-site	1	1	1			2			1		

Figure S11. Analysis of *cis*-acting elements of MbLOX gene family promoter.

Category	Function	Element	MiLOX15	MiLOX6	MiLOX13	MiLOX18	MiLOX12	MiLOX7	MiLOX14	MiLOX5	MiLOX1	MiLOX4	MiLOX16	MiLOX10
light responsive	light	Box 4	1	1	2	1	6			1	3		2	1
		GATA-motif	1	2				1	2	1		1	1	
		G-box	12	2	4	1	6	9	18		1	7	3	2
		GT1-motif		2	1	4					1	2	2	
		MRE	2					1	1	1	1	1		
		Sp1				1	1		1	1				4
		TCCC-motif	1	1		1		1		1			1	
		TCT-motif	1			1	4	1	1					
phytohormone responsive	Abscisic acid	ABRE	8	1	3	1	7	8	16			6	3	2
	Auxin	AuxRR-core							1		1			
		TGA-element	1		3			1						
	MeJA	CGTCA-motif	1			1	1	3	1	1		1	1	1
		TGACG-motif	1			1	1	3	1	1		1	1	1
	gibberellin	P-box		1	3									
		TATC-box		1	1							1		
		GARE-motif								1				
	salicylic acid	TCA-element						1		1				
	Ethylene	ERE				1	2	3	5					
stress responsive	anaerobic induction	ARE	3	1		1	1	2	1	2	2	1		2
	low-temperature	LTR	1			1		3	1	2				
	MYB-drought	MBS		1	1		1			2				
	defense and stress	TC-rich repeats							1		1		1	
	Wound	WUN-motif		1		1						1		2
plant growth and development	meristem	CAT-box			1	1	1				1			
	circadian control	circadian								1	2			
	endosperm	GCN4_motif		1		1		1		1				
	zein metabolism	O2-site			1		1			4	1	1		

Figure S12. Analysis of *cis*-acting elements of MiLOX gene family promoter.

A

	MbLOX8	MbLOX10	MbLOX11	MbLOX9	MbLOX16	MbLOX1	MbLOX14	MbLOX18	MbLOX15	MbLOX6	MbLOX13
AP2/ERF		2		1	8	2		5	4	2	
ARF		1		1							
BBR-BPC						17		1			
C2H2											
Dof	1	5		1	5	3		4	1		
GATA											2
MIKC_MADS		5			9	1		10	1		
MYB	2		1			1					

B

	MiLOX15	MiLOX6	MiLOX13	MiLOX18	MiLOX12	MiLOX7	MiLOX14	MiLOX5	MiLOX1	MiLOX4	MiLOX16	MiLOX10
AP2/ERF		2	13			1	2			2	2	
BBR-BPC			36						4			
C2H2				1	3							
Dof	4				3	3					1	1
GATA				1	6							
MIKC_MADS			12			6	4			1	3	3
MYB			1							1		1
TALE	1		11									

Figure S13. Transcription factor binding sites predicted in the promoters of *MbLOXs* (A) and *MiLOXs* (B).

Arg(R)	CGC **1.30**	AGG **1.61**	CGG **1.08**	AGA 0.82	CGA 0.68	CGU 0.51
Leu(L)	CUG **2.00**	CUC **1.98**	UUG 0.97	CUU 0.69	CUA 0.25	UUA 0.11
Ser(S)	AGC **2.07**	UCC **1.46**	UCG **1.18**	AGU 0.50	UCU 0.50	UCA 0.30
Ala(A)	GCC **1.61**	GCG **1.27**	GCU 0.57	GCA 0.55	- 0	- 0
Gly(G)	GGC **1.99**	GGG 0.91	GGA 0.71	GGU 0.39	- 0	- 0
Pro(P)	CCG **1.35**	CCC **1.21**	CCU 0.81	CCA 0.63	- 0	- 0
Thr(T)	ACC **1.71**	ACG **1.38**	ACA 0.49	ACU 0.42	- 0	- 0
Val(V)	GUC **1.67**	GUG **1.65**	GUU 0.48	GUA 0.20	- 0	- 0
Ile(I)	AUC **2.33**	AUA 0.38	AUU 0.29	- 0	- 0	- 0
Asn(N)	AAC **1.48**	AAU 0.52	- 0	- 0	- 0	- 0
Asp(D)	GAC **1.35**	GAU 0.65	- 0	- 0	- 0	- 0
Cys(C)	UGC **1.63**	UGU 0.37	- 0	- 0	- 0	- 0
Gln(Q)	CAG **1.43**	CAA 0.58	- 0	- 0	- 0	- 0
Glu(E)	GAG **1.60**	GAA 0.40	- 0	- 0	- 0	- 0
His(H)	CAC **1.44**	CAU 0.56	- 0	- 0	- 0	- 0
Lys(K)	AAG **1.72**	AAA 0.28	- 0	- 0	- 0	- 0
Phe(F)	UUC **1.75**	UUU 0.25	- 0	- 0	- 0	- 0
Tyr(Y)	UAC **1.63**	UAU 0.37	- 0	- 0	- 0	- 0
Trp(W)	UGG 1.00	- 0	- 0	- 0	- 0	- 0
Met(M)	AUG 1.00	- 0	- 0	- 0	- 0	- 0

Figure S14. Relative usage of synonymous codons in MbLOX gene family members. The underlined data indicate that the MbLOX genes preferentially to use this codon.

Arg(R)	AGG **1.69**	CGC **1.19**	CGG **1.14**	AGA 0.84	CGA 0.70	CGU 0.43
Leu(L)	CUG **1.93**	CUC **1.90**	UUG 0.95	CUU 0.72	CUA 0.34	UUA 0.16
Ser(S)	AGC **1.80**	UCC **1.51**	UCG **1.12**	AGU 0.57	UCU 0.55	UCA 0.46
Ala(A)	GCC **1.60**	GCG **1.25**	GCU 0.70	GCA 0.45	- 0	- 0
Gly(G)	GGC **1.84**	GGG 0.92	GGA 0.82	GGU 0.42	- 0	- 0
Pro(P)	CCG **1.42**	CCC **1.04**	CCU 0.80	CCA 0.75	- 0	- 0
Thr(T)	ACC **1.54**	ACG **1.36**	ACU 0.56	ACA 0.55	- 0	- 0
Val(V)	GUC **1.68**	GUG **1.65**	GUU 0.43	GUA 0.24	- 0	- 0
Ile(I)	AUC **2.23**	AUA 0.45	AUU 0.32	- 0	- 0	- 0
Asn(N)	AAC **1.42**	AAU 0.58	- 0	- 0	- 0	- 0
Asp(D)	GAC **1.33**	GAU 0.67	- 0	- 0	- 0	- 0
Cys(C)	UGC **1.67**	UGU 0.33	- 0	- 0	- 0	- 0
Gln(Q)	CAG **1.46**	CAA 0.54	- 0	- 0	- 0	- 0
Glu(E)	GAG **1.54**	GAA 0.46	- 0	- 0	- 0	- 0
His(H)	CAC **1.30**	CAU 0.70	- 0	- 0	- 0	- 0
Lys(K)	AAG **1.63**	AAA 0.37	- 0	- 0	- 0	- 0
Phe(F)	UUC **1.64**	UUU 0.36	- 0	- 0	- 0	- 0
Tyr(Y)	UAC **1.57**	UAU 0.43	- 0	- 0	- 0	- 0
Trp(W)	UGG 1.00	- 0	- 0	- 0	- 0	- 0
Met(M)	AUG 1.00	- 0	- 0	- 0	- 0	- 0

Figure S15. Relative usage of synonymous codons in MiLOX gene family members. The underlined data indicate that the MiLOX genes preferentially to use this codon.
